# Diagnostic Considerations for Neurolymphomatosis: A Natural History Analysis

**DOI:** 10.3390/cancers18132068

**Published:** 2026-06-25

**Authors:** Francesca Rothell, Mary Ann Nguyen, Elizabeth Xu, Quan Ho, Sibo Zhou, Shiva Gautam, Eric T. Wong

**Affiliations:** 1 Division of Hematology/Oncology, Brown University Health Cancer Institute, Rhode Island Hospital, The Warren Alpert Medical School, Brown University, Providence, RI 02903, USA; frothell@brownhealth.org; 2 Faculty of Computing & Data Sciences, Boston University, Boston, MA 02215, USA; 3 Department of Chemistry, University of Pennsylvania, Philadelphia, PA 19104, USA; 4 Department of Biomedical Engineering, Boston University, Boston, MA 02215, USA; 5 Data Science Institute, Brown University, Providence, RI 02906, USA; sibo_zhou@alumni.brown.edu; 6 Department of Biostatistics, University of Florida, Gainesville, FL 32611, USA

**Keywords:** lymphoma, peripheral nerves, imaging, biopsy

## Abstract

Neurolymphomatosis (NL), a rare manifestation of non-Hodgkin’s lymphoma affecting the peripheral nervous system, remains a diagnostic challenge. This study aimed to define an optimal diagnostic approach for timely and effective identification of NL. We analyzed 559 NL cases from 231 articles published from 1951 to 2022, examining how patient outcomes correlated with various diagnostic modalities, including magnetic resonance imaging (MRI), computed tomography (CT), [^18^F]fluorodeoxyglucose positron emission tomography (FDG-PET), electromyography-nerve conduction studies (EMG-NCS), ultrasound, and tissue biopsy when used individually or in combination. Separate analyses were performed in a mutually exclusive fashion to minimize confounding effects from multiple modalities. The results of this investigation revealed that patients with biopsies had a longer time interval from first treatment to progression (Kruskal–Wallis *p* < 0.0001), survival from diagnosis (overall survival) (*p* < 0.0001), and survival from symptom onset (*p* < 0.0001), but not symptom onset to diagnosis (*p* = 0.2134). Pairwise comparisons of biopsy plus 2, 3, or 4 diagnostic modalities revealed a positive trend for the combination of biopsy + PET + MRI + EMG-NCS. A majority of patients without biopsy had secondary NL. In this non-biopsied population, no diagnostic modality had a significant correlation with outcome. The data collectively indicate that histological confirmation of NL from biopsy was associated with a positive patient outcome. Management of NL patients requires timely testing using PET, MRI, and EMG-NCS to quickly identify a site for image-guided nerve biopsy.

## Simple Summary

Diagnosis of lymphoma involving peripheral nerves, also known as neurolymphomatosis (NL), is challenging. Although responsive to treatment, this malignancy is often not identified until after death of the patient during autopsy. By analyzing 559 neurolymphomatosis cases from 231 publications, we queried whether a combination of studies can help with timely diagnosis. We found that those patients who had biopsy of nothing other than affected peripheral nerves (also known as primary NL) lived longer, while others with a prior diagnosis of lymphoma elsewhere (identified as secondary NL) often did not undergo a biopsy. A combination of positron emission tomography, magnetic resonance imaging and electromyography-nerve conduction study can quickly identify a site for image-guided nerve biopsy. This strategy most likely enables timely diagnosis and earlier intervention in patients with suspected NL.

## 1. Introduction

Neurolymphomatosis (NL), an extranodal lymphoma that invades the peripheral nervous system, remains a diagnostic challenge, and timely diagnosis is a major limiting factor to treatment [1,2,3,4,5,6,7,8]. The predominant histology in humans is CD20+ B-cell non-Hodgkin’s lymphoma with a proliferation index over 90% [9]. Patients tend to present clinically in one of four patterns: (i) painful involvement of nerve roots, (ii) cranial neuropathy with or without pain, (iii) painless involvement of peripheral nerves, or (iv) painful or painless involvement of a single peripheral nerve [10]. Although advances in treatment have led to prolonged survival in this population, the diagnosis of many are established at post-mortem [1,11,12,13,14,15]. Optimal diagnostic measures for NL remain unknown.

A prior natural history study of NL patients by Xu et al., which explored prognostic factors and outcome characteristics from case reports and series published from 1951 to 2022, identified that there was progressive lengthening of survival and time to progression [1]. The most important finding was a deficiency in timely diagnosis, as the interval from symptom onset to diagnosis remained flat over successive decades. Diagnosis remains a major obstacle to efficacious treatment for patients with NL. Therefore, we performed this investigation, building on the prior dataset collected by Xu et al. [1], and added various diagnostic modalities used in establishing NL. We found that biopsy was associated with a positive influence on patient outcome. The role for other diagnostic modalities is to quickly pinpoint a site amenable for image-guided biopsy.

## 2. Methods

**Figure 1:**
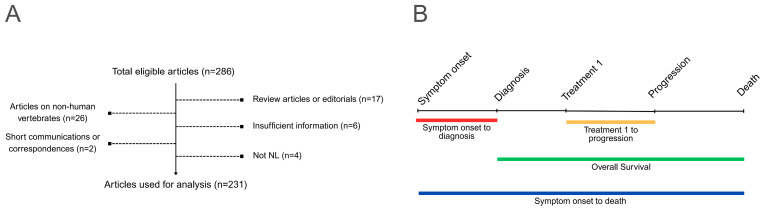
Consolidated Standards of Reporting Trials (CONSORT) diagram and graphical depiction of outcome parameters. (**A**) CONSORT diagram for article inclusion and exclusion. (**B**) Outcome parameters include (i) time from symptom onset to diagnosis (red), (ii) time from treatment 1 to progression (gold), (iii) survival from diagnosis to death (green), and (iv) survival from symptom onset to death (blue).

We used the dataset from prior natural history analysis of NL by Xu et al., and it consisted of 559 NL patient cases from 231 articles published from 1851 to 2022 [1] (Figure 1A and Supplementary Table S16). Additional data related to diagnostic modalities were collected from each patient, including reports of magnetic resonance imaging (MRI), computed tomography (CT), [^18^F]fluorodeoxyglucose positron emission tomography (FDG-PET), electromyography-nerve conduction study (EMG-NCS), and tissue biopsy. Furthermore, patients were subcategorized as having primary NL, when the malignancy is found only in the nervous system, or with secondary NL, having had a prior diagnosis of systemic lymphoma that subsequently infiltrated into the nervous system. Missing data were not imputed in the final analysis due to heterogeneity of published information. In cases where aggregate statistics were reported, providing a proportion of patients who had any given imaging diagnostic test performed, the authors chose to deconvolute the data to create a collective dataset representing individual cases.

Statistical analyses were performed in R version 4.2.3 using packages: ggplot2, plyr, survival, survminer, prodlim, Hmisc, pracma, readxl and ggpubr. To compare the impact of various diagnostic modalities used, the same survival metrics were utilized as in the original study, including (i) time from symptom onset to diagnosis, (ii) time from treatment 1 (first treatment) to progression, (iii) survival from diagnosis to death (overall survival), and (iv) survival from symptom onset to death (Figure 1B).

Kruskal–Wallis test was performed to determine differences among groups, with Bonferroni adjustment to minimize false positives from multiple testing. Statistical significance was defined at *p* ≤ 0.05.

## 3. Results

### 3.1. Patients

The median age was 61 (range 2–92) years. There were 326 males, 222 females, and 11 patients of unspecified sex. Adults at age ≥ 18 years comprised the majority of the NL population (n = 541), while a few had unspecified ages (n = 13) or were adolescents from age 13 to 17 (n = 3), child at age 8 (n = 1), and toddler at age 2 (n = 1). CONSORT diagram and the timeline for outcome parameters used in our analysis are described in Figure 1A and Figure 1B, respectively. Patient survival, sites of peripheral nerve involvement, histological characteristics of NL, and treatment outcome have been reported previously [1]. Treatments used include (i) corticosteroid alone, (ii) high-dose methotrexate alone, (iii) rituximab-based therapies, (iv) radiation alone, (v) high-dose methotrexate + rituximab-based treatment, (vi) transplant, (vii) high-dose methotrexate + transplant, (viii) rituximab-based therapy + transplant, and (ix) high-dose methotrexate + rituximab-based therapy + transplant [1].

### 3.2. Comparison Among Different Diagnostic Modalities in NL

**Figure 2:**
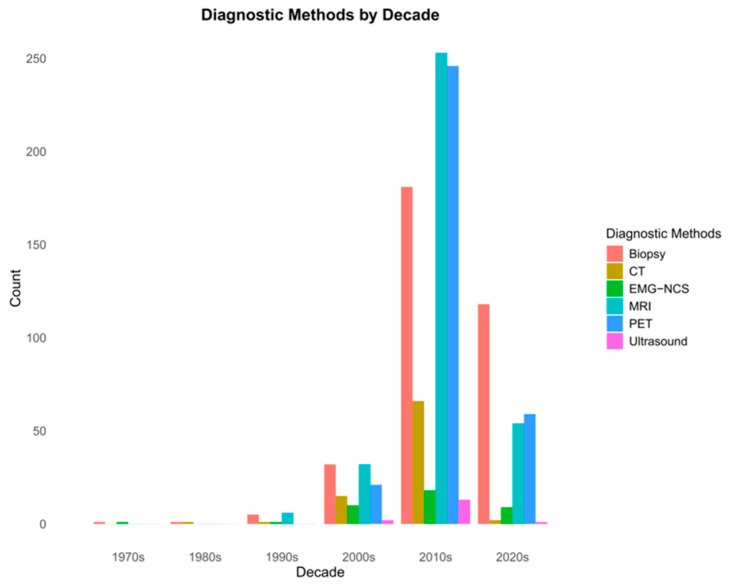
Utilization frequency of diagnostic modalities in successive decades.

We first compared various diagnostic modalities used in the NL population, including biopsy, MRI, CT, PET, EMG-NCS, and ultrasound. There is an increased utilization of these diagnostic modalities over the decades (Figure 2).

**Figure 3:**
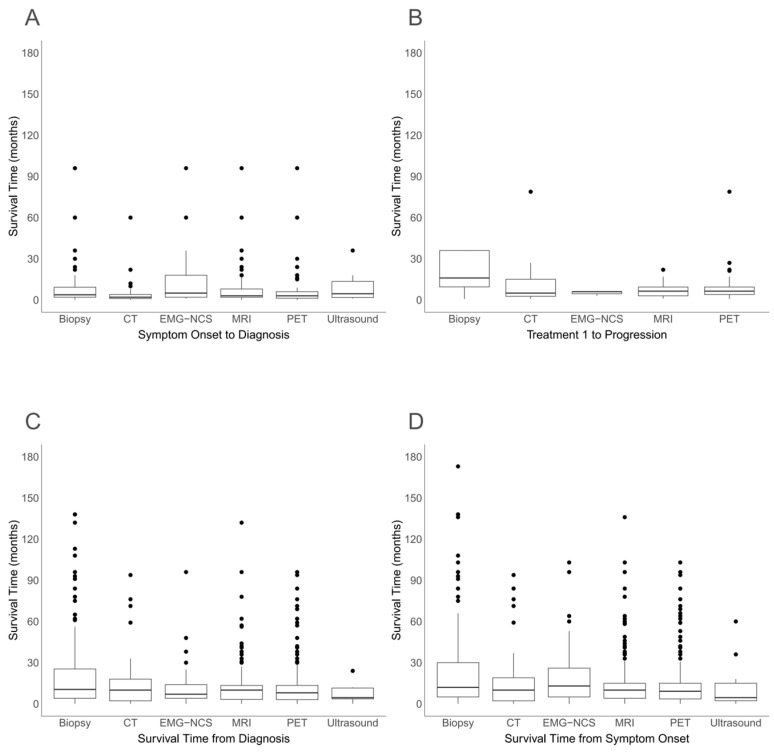
NL patient survival associated with various diagnostic modalities (non-mutually exclusive). Box-and-whisker plot of survival times according to biopsy, CT, EMG-NCS, MRI, PET and ultrasound with respect to time period from (**A**) symptom onset to diagnosis (Kruskal–Wallis *p* = 0.2135), (**B**) treatment 1 to progression (*p* < 0.0001), (**C**) survival time from diagnosis until death (*p* < 0.0001), and (**D**) survival time from symptom onset until death (*p* < 0.0001).

We found that patients who had biopsies appeared to have a longer survival time from treatment 1 to progression (Figure 3B and Supplementary Table S1B, Kruskal–Wallis *p* < 0.0001), survival from diagnosis (Figure 3C and Supplementary Table S1C, *p* < 0.0001), and survival from symptom onset (Figure 3D and Supplementary Table S1D, *p* < 0.0001), but not from symptom onset to diagnosis (Figure 3A and Supplementary Table S1A, *p* = 0.2135).

**Figure 4:**
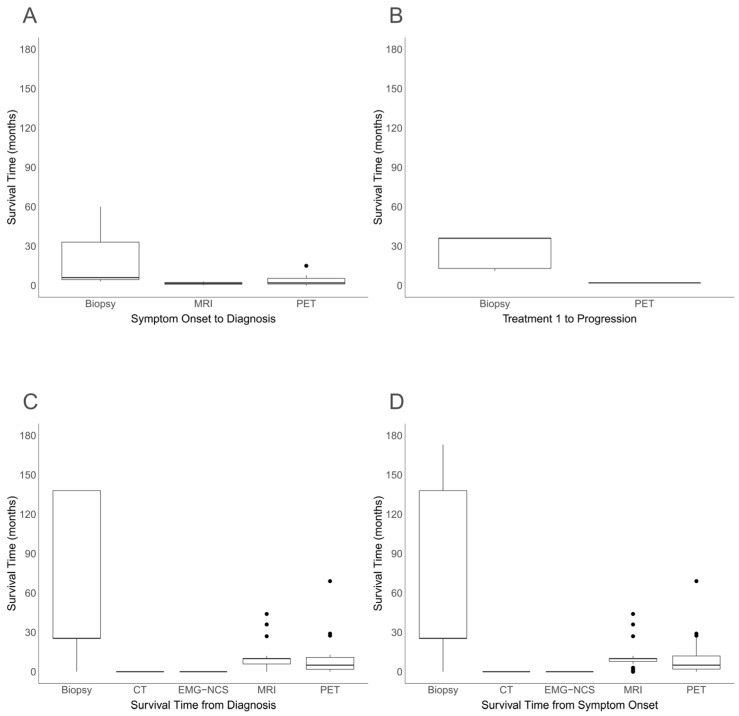
NL patient survival associated with various diagnostic modalities (mutually exclusive). Box-and-whisker plot of survival times according to biopsy, CT, EMG-NCS, MRI, PET and ultrasound with respect to time period from (**A**) symptom onset to diagnosis (Kruskal–Wallis *p* = 0.2555), (**B**) treatment 1 to progression (N/A), (**C**) survival time from diagnosis (*p* < 0.0001), and (**D**) survival time from symptom onset (*p* < 0.0001).

Furthermore, biopsy (n = 248) appears to be associated with an improvement in survival compared to MRI (n = 276) or PET (n = 236), but the lack of significance with CT (n = 82), EMG-NCS (n = 37), and ultrasound (n = 14) may be a result of their smaller sample size (Supplementary Table S1D). Because a majority of patients underwent multiple diagnostic studies, we therefore conducted a separate analysis comparing each modality when performed in a mutually exclusive fashion. This analysis still showed that biopsy led to a longer survival from diagnosis (Figure 4C and Supplementary Table S2C, *p* < 0.0001) and survival from symptom onset (Figure 4D and Supplementary Table S2D, *p* < 0.0001) but not time from symptom onset to diagnosis (Figure 4A and Supplementary Table S2A, *p* = 0.2555), while treatment 1 to progression lacked statistical power (Figure 4B and Supplementary Table S2B). These findings indicate that biopsy was associated with better survival measures in our NL patients, but it did not necessarily lead to a timely diagnosis or treatment outcome.

### 3.3. Patients Who Underwent Biopsies Lived Longer

**Figure 5:**
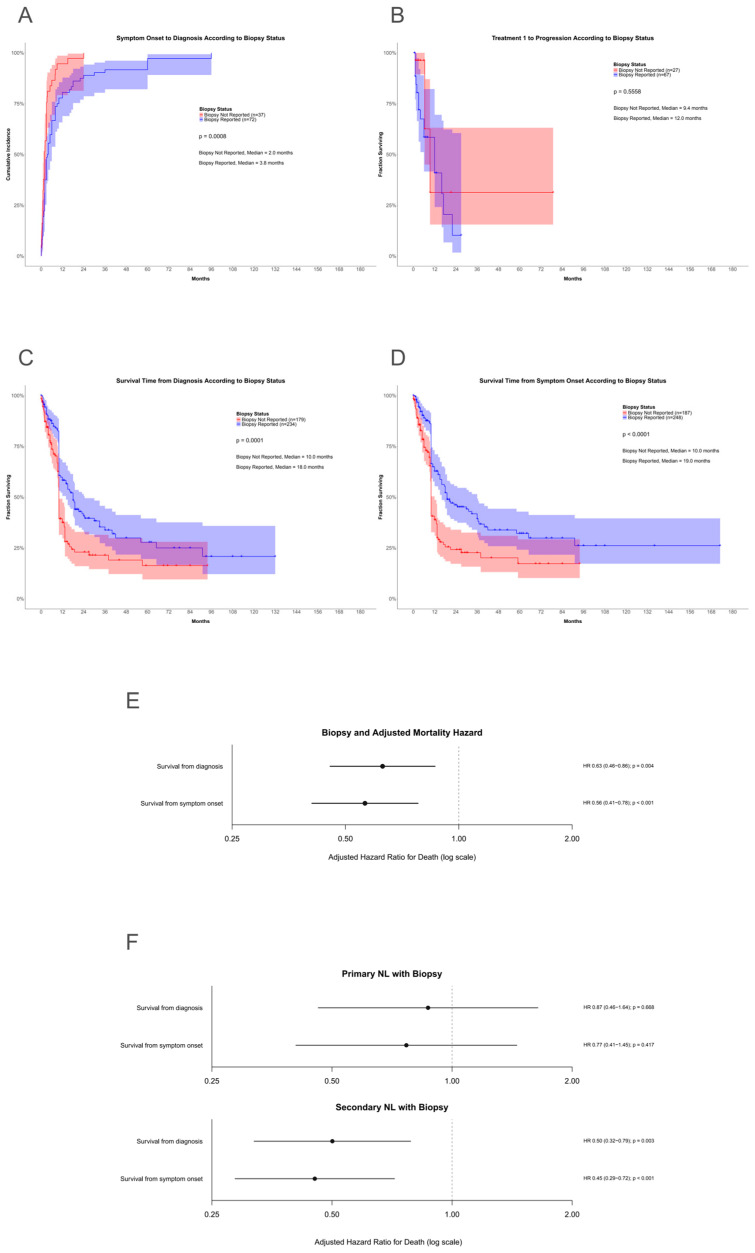
Kaplan–Meier survival for NL patients stratified according to presence or absence of biopsy. Patients with and without biopsy had a (**A**) respective median time from symptom onset to diagnosis of 3.8 (95%CI 3.0–6.0) and 2.0 (95%CI 1.3–3.0) months (*p* = 0.0008), (**B**) respective median time from treatment 1 to progression of 12.0 (95%CI 6.0-NA) and 9.4 (95%CI 6.4-NA) months, (*p* = 0.5558), (**C**) respective median survival from diagnosis of 18.0 (95%CI 14.0–24.5) and 10.0 (95%CI 10.0–10.0) months (*p* = 0.0001), and (**D**) respective median survival from symptom onset of 19.0 (95%CI 16.0–36.0) and 10.0 (95%CI 10.0–12.0) months (*p* < 0.0001). (**E**) Cox proportional hazard analysis showed that biopsy was associated with decreased hazard ratio with respect to survival from diagnosis (HR = 0.63 (95%CI 0.46–0.86), *p* = 0.004) and survival from symptom onset (HR = 0.56 (95%CI 0.41–0.78), *p* < 0.001). (**F**) Biopsy was not associated with a decreased hazard ratio in primary NL with respect to survival from diagnosis (HR = 0.87 (95%CI 0.46–1.64), *p* = 0.668) or survival from symptom onset (HR = 0.77 (95%CI 0.41–1.45), *p* = 0.417). But biopsy lowered the hazard ratio in secondary NL with respect to survival from diagnosis (HR = 0.50 (95%CI 0.32–0.79), *p* = 0.003) and survival from symptom onset (HR = 0.45 (95%CI 0.29–0.72), *p* < 0.001).

We then performed Kaplan–Meier statistics on our cohort comparing patients with and without biopsy for histological diagnosis of NL. As expected, those with a biopsy had a longer survival from diagnosis (Figure 5C) and survival from symptom onset (Figure 5D). However, contrary to our expectation, patients with or without a biopsy had a similar median time from symptom onset to diagnosis, and nearly all of those without a biopsy were diagnosed within the first 24 months of symptom onset. Of note, within the biopsied cohort, there were seven patients with an exceptionally long period (36 to 96 months) from symptom onset to diagnosis (Figure 5A). Furthermore, treatment did not make a statistical difference between these two groups (Figure 5B). However, multivariate Cox proportional hazard analysis showed biopsy was associated with a decreased hazard for mortality (Figure 5E). Interestingly, decreased hazard ratio was seen in patients with secondary but not primary NL (Figure 5F).

**Figure 6:**
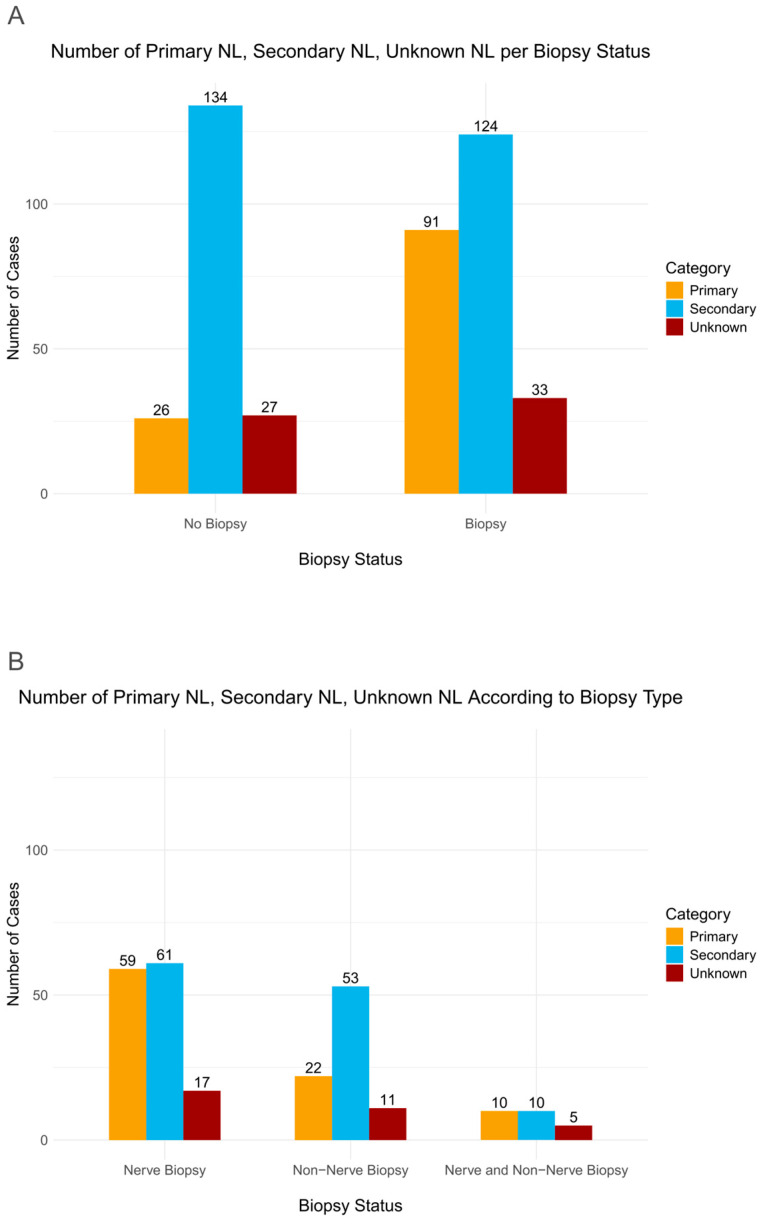
Number of primary versus secondary NL patients with and without biopsy. (**A**) Five times more secondary NL patients (n = 134) did not have a biopsy compared to those with primary NL (n = 26). Among those with biopsies, this difference is only 1.4 times between secondary (n = 124) and primary (n = 91) NL patients. (**B**) Similar numbers of secondary (n = 61) and primary (n = 59) NL patients had nerve biopsy, but more than a 2-fold difference was seen in non-nerve biopsy between secondary (n = 53) and primary (n = 22) NL. The same number of primary (n = 10) and secondary (n = 10) NL patients had both nerve and non-nerve biopsies.

These findings prompted us to investigate the origin of NL underlying these two groups. Indeed, patients without biopsy were more likely to have secondary than primary NL (n = 134 vs. 26, respectively) compared to those with biopsies (n = 124 vs. 91, respectively) (Figure 6A). We next asked whether there is a difference in the number of patients who underwent nerve versus non-nerve biopsies in the biopsied population. The number of nerve biopsies were equivalent between secondary and primary NL (n = 61 vs. 59, respectively) while non-nerve biopsies were numerically different (n = 53 vs. 22, respectively) (Figure 6B). A smaller number had both nerve and non-nerve biopsies (n = 10 for both groups) (Figure 6B).

With the higher proportion of non-biopsied patients who were diagnosed with secondary NL, our data indicate that these patients were likely diagnosed earlier compared to those with primary NL. However, this earlier diagnosis did not translate into better treatment outcome. We also asked whether there were differences in diagnosis, treatment, and survival among patients having nerve biopsy, non-nerve biopsy, or both. There was no difference in the three groups with respect to symptom onset to diagnosis (*p* = 0.3820, Supplementary Figure S1A), treatment 1 to progression (*p* = 0.4742, Supplementary Figure S1B), or survival from diagnosis (*p* = 0.1298, Supplementary Figure S1C). There was only a trend for better survival from symptom onset in those with non-nerve biopsies (*p* = 0.0722, Supplementary Figure S1D).

### 3.4. Association Between Survival and Diagnostic Modalities

We next investigated the association between survival and a combination of biopsy with another diagnostic modality. No combination stood out among biopsy + MRI, PET, CT, ultrasound, or EMG-NCS in the periods from symptom onset to diagnosis (*p* = 0.4798), treatment 1 to progression (*p* = 0.8888), survival from diagnosis (*p* = 0.8360), or survival from symptom onset (*p* = 0.3936) (Supplementary Tables S3). In the analysis for survival from symptom onset, pairwise comparison showed that the combination of biopsy + EMG-NCS had a longer duration than biopsy + ultrasound, but the significance markedly disappeared when adjusted by Bonferroni correction. The lack of statistical significance in these combinations may be due to the overwhelming influence of biopsy on survival. Therefore, biopsy combined with another diagnostic modality offers no additional benefit compared to biopsy itself. Furthermore, to remove the confounding effect of multiple diagnostic studies in the same patient, we conducted a separate analysis in a mutually exclusive fashion. This analysis also showed no significance in symptom onset to diagnosis (*p* = 0.9548), treatment 1 to progression (*p* = 1.0000), survival from diagnosis (*p* = 0.9414), or survival from symptom onset (*p* = 0.9801) (Supplementary Table S4).

We further investigated whether biopsy + 2, 3, or 4 other diagnostic modalities would yield a combination that is statistically significant. First, biopsy + 2 additional modalities were not significant for the periods from symptom onset to diagnosis (*p* = 0.2226), treatment 1 to progression (*p* = 0.7868), or survival from diagnosis (*p* = 0.2586), except for survival from symptom onset (*p* = 0.0410) (Supplementary Table S5). The only comparison that showed significance (*p* = 0.0236) after Bonferroni correction was biopsy + PET + EMG-NCS as being superior to biopsy + PET + ultrasound, suggesting that EMG-NCS could potentially add value when combined with other diagnostic modalities. Second, biopsy + 3 additional modalities was not significant for the periods from symptom onset to diagnosis (*p* = 0.4039) or survival from diagnosis (*p* = 0.0828), except for survival from symptom onset (*p* = 0.00331), while treatment 1 to progression did not have enough statistical power for Kruskal–Wallis analysis (Supplementary Figure S2 and Supplementary Table S6). In both cohorts of survival from diagnosis and from symptom onset, the comparison showed a trend favoring biopsy + PET + MRI + EMG-NCS over biopsy + PET + MRI + ultrasound (*p* = 0.0779 and *p* = 0.0962, respectively). These findings further suggest greater utility of EMG-NCS over ultrasound, albeit a minor advantage. Lastly, there were not enough data points for statistical analysis of biopsy + 4 additional modalities (Supplementary Table S7). Mutually exclusive Kruskal–Wallis analysis of biopsy + 2 or 3 additional modalities (Supplementary Tables S8 and S9; Supplementary Figure S3) only revealed significance (*p* = 0.0082) in biopsy + PET + MRI + EMG-NCS for survival from symptom onset (Supplementary Table S9D).

A significant number of patients did not have a biopsy. Therefore, we asked whether there is an individual diagnostic modality that correlates with timely diagnosis or survival advantage. None were statistically significant for any of the four time periods (Supplementary Table S10). We then investigated timely diagnosis and survival advantage from a combination of two or three diagnostic modalities. Still, none was statistically significant (Supplementary Tables S11 and S12). Furthermore, mutually exclusive Kruskal–Wallis analyses of individual or a combination of 2 or 3 diagnostic modalities also did not show significance (Supplementary Tables S13–S15). These findings suggest that no diagnostic imaging technique, either alone or in combination, correlates with survival in patients without biopsy.

## 4. Discussion

A major finding from our analysis is the significance of biopsy in NL patients. Biopsied patients had longer survival from symptom onset and longer survival from time of diagnosis. Although current research strives to identify the most efficacious treatment regimen for NL, this focus may be short-sighted because half of the population was not diagnosed in a timely fashion to even be considered for treatment [1,16,17,18]. Our work indicates that biopsy is associated with better outcomes and a focus on achieving biopsies more efficiently may be worthwhile.

The site selected for biopsy requires guidance by neuroimaging, neurophysiologic study, or both. None of the individual modalities tested, including MRI, PET, CT, EMG-NCS, and ultrasound, had an overwhelming impact on patient outcomes when combined with biopsy. Therefore, we investigated the utilization of multi-modality diagnostic techniques in this setting. Our Kruskal–Wallis analyses, with correction for multiple testing using the most stringent Bonferroni adjustment, revealed that biopsy + PET + EMG-NCS with or without MRI appears to be the best combination of modalities associated with longest survival. Because NL patients often underwent multiple or even all of the diagnostic studies examined, we performed additional analyses to determine only the mutually exclusive effect of one or more modalities per patient that can guide biopsy. Still, biopsy + PET + MRI + EMG-NCS appears to be the best combination after Bonferroni adjustment. Together, the diagnosis of NL patients may require timely testing using PET, MRI, and EMG-NCS to quickly identify a site for image-guided nerve biopsy so that they can proceed to treatment.

The distribution of NL subtypes is different in patients with and without biopsy. Compared to those with biopsy, patients without biopsy predominantly have secondary NL (secondary vs. primary NL: 124 vs. 91 with biopsy and 134 vs. 26 without biopsy, respectively). This is probably due to secondary NL patients being more likely to forgo a biopsy, given their pre-existing lymphoma diagnosis. In some reports, biopsies were also specifically mentioned not to have been performed where a nerve biopsy would have posed too much risk for injury. Our multivariate analysis showed that biopsy lowered the hazard ratio in secondary but not primary NL. This may be a result of an imbalance in the cohort with primary NL skewed toward biopsy, while the numbers are more evenly distributed in secondary NL. Furthermore, biopsy outside the nervous system is also more frequently seen in secondary NL (secondary vs. primary NL: 53 vs. 22), most likely to confirm the recurrence of systemic lymphoma. Unfortunately for this population, no diagnostic modality, either alone or in combination, had a significant correlation with outcome.

Prior analysis of NL revealed that aggressive systemic treatment is associated with a slightly longer overall survival and progression-free survival compared to local therapy, such as radiation [1]. This suggests that NL should be viewed as a systemic malignancy rather than a localized disease. Indeed, a majority of NL patients were treated with high-dose methotrexate or methotrexate-based regimen with or without rituximab [1]. Other treatments include Bruton’s tyrosine kinase inhibitor, gene therapy, autologous stem cell transplantation, bispecific antibody therapy, chimeric antigen receptor T-cell therapy or other immunotherapies [19,20,21,22,23]. As expected, we found no difference in treatment outcome when the population was dichotomized into biopsied and non-biopsied cohorts, most likely because NL patients have a poor outcome in general, and both cohorts were treated with systemic chemotherapy. However, we also noted that more secondary NL patients received empirical treatment without biopsy for pathological confirmation.

Cerebral intravascular lymphomatosis (IVL) is another aggressive, extranodal non-Hodgkin’s lymphoma that is difficult to diagnose in a timely fashion [24]. Like NL, half of the population of IVL is diagnosed post-mortem, and time from symptom onset to diagnosis is unclear [1,25]. However, the lymphoma cells in IVL have a predilection for vascular endothelium, likely due to a lack of β1-integrin and ICAM-1, which prevents diapedesis and infiltration into the brain [26]. Therefore, IVL is primarily a malignancy in the systemic circulation while NL involves both sides of the blood–nerve barrier [27]. This may be the reason that IVL often appears in other systemic sites, particularly in skin, far more frequently than in bone marrow, spleen, and the lungs [25]. A diagnostic skin biopsy has definitely far less risk compared to biopsies at other systemic sites [28]. Although NL does not appear to involve the skin at least by gross inspection, microscopic infiltration into the dermis or epidermis is possible. Skin biopsy has been used for diagnosis of small fiber peripheral neuropathy, and this minimally invasive technique may be of value to patients with NL, guided by neuroimaging, EMG-NCS, or both [29].

Our work is limited by the heterogeneity in data extracted from case reports, small patient series, and the retrospective analysis from publications. However, NL is a rare malignancy involving the peripheral nervous system, which precludes prospective data collection for a natural history study. Examination of the existing literature in aggregate is the only means of a comprehensive analysis of this disorder. Furthermore, we chose to deconvolute the aggregate statistics from three larger series into individual patient data rather than treating each publication as an individual data point to develop a more comparable dataset [16,17,30]. In addition, a few cases of lymphoma- or lymphoid-like leukemic NL were included because they were difficult to differentiate from lymphoma or leukemia. But cases that only reported neuroimaging or neuropathology findings without any clinical data from patients were excluded. We believe that these approaches will help define the optimal diagnostic modality for NL. Indeed, despite the above limitations, we were able to identify that biopsy for histologic diagnosis correlates with a favorable outcome for NL patients, and MRI + PET + EMG-NCS are the initial diagnostic modalities in combination that can help quickly identify an affected site appropriate for biopsy. Importantly, those who are successfully diagnosed in a timely fashion and treated may have better overall health status, access to specialized care, or both. Validation will be needed, however, from an open-label study in the real world or from another large-scale retrospective dataset.

## 5. Conclusions

It is difficult to diagnose NL, and our analysis showed that timely diagnosis of this malignancy makes a difference in patient outcome. MRI, PET, and EMG-NCS in combination are probably the optimal diagnostic studies that help oncologists in selecting a site for image-guided nerve biopsy.

## Data Availability

The original contributions presented in the study are included in the article/Supplementary Material. Further inquiries can be directed to the corresponding author.

## Supplementary Materials

The following supporting information can be downloaded at: https://www.mdpi.com/article/10.3390/cancers18132068/s1, Figure S1: Kaplan–Meier survival for NL patients with nerve biopsy only, non-nerve biopsy only, or both. Patients with nerve, non-nerve or both types of biopsy had a (A) respective median time from symptom onset to diagnosis of 6.0 (95%CI 3.0–10.0), 3.0 (95%CI 2.0–6.0) and 3.3 (95%CI 2.0–NA) months (*p* = 0.3820), (B) respective median time from treatment 1 to progression of 22.0 (95%CI 3.0–NA), 12.0 (95%CI 2.0–NA) and 12.0 (95%CI 4.0–NA) months, (*p* = 0.4742), (C) respective median survival from diagnosis of 14.0 (95%CI 10.0–24.5), 24.0 (95%CI 18.5–NA) and 15.0 (95%CI 11.0–NA) months (*p* = 0.1298), and (D) respective median survival from symptom onset of 16.0 (95%CI 10.0–33.0), not reach (95%CI 19.0–NA) and 15.0 (95%CI 11.0–NA) months (*p* = 0.0722); Figure S2: NL patient survival associated with biopsy +3 additional diagnostic modalities (non-mutually exclusive). Box-and-whisker plot of survival times according to biopsy plus a combination of 3 of the following diagnostic modalities: CT, EMG-NCS, MRI, PET and ultrasound with respect to time period from (A) symptom onset to diagnosis (Kruskal–Wallis *p* = 0.4039), (B) treatment 1 to progression (N/A), (C) survival time from diagnosis (*p* = 0.0828), and (D) survival time from symptom onset (*p* = 0.0331); Figure S3: NL patient survival associated with biopsy +3 additional diagnostic modalities (non-mutually exclusive). Box-and-whisker plot of survival times according to biopsy plus a combination of 3 of the following diagnostic modalities: CT, EMG-NCS, MRI, PET and ultrasound with respect to time period from (A) symptom onset to diagnosis (Kruskal–Wallis *p* = 0.4039), (B) treatment 1 to progression (N/A), (C) survival time from diagnosis (*p* = 0.0828), and (D) survival time from symptom onset (*p* = 0.0331); Table S1: Kruskal–Wallis analysis of individual diagnostic modalities (non-mutually exclusive) with respect to (A) Time From Symptom Onset to Diagnosis, (B) Time From Treatment 1 to Progression, (C) Survival Time from Diagnosis, (D) Survival Time from Symptom Onset; Table S2: Kruskal–Wallis analysis of individual diagnostic modalities (mutually exclusive) with respect to (A) Time From Symptom Onset to Diagnosis, (B) Time From Treatment 1 to Progression, (C) Survival Time from Diagnosis, (D) Survival Time from Symptom Onset; Table S3: Kruskal–Wallis analysis of biopsy +1 diagnostic modalities (non-mutually exclusive) with respect to (A) Time From Symptom Onset to Diagnosis, (B) Time From Treatment 1 to Progression, (C) Survival Time from Diagnosis, (D) Survival Time from Symptom Onset; Table S4: Kruskal–Wallis analysis of biopsy +1 diagnostic modalities (mutually exclusive) with respect to (A) Time From Symptom Onset to Diagnosis, (B) Time From Treatment 1 to Progression, (C) Survival Time from Diagnosis, (D) Survival Time from Symptom Onset; Table S5: Kruskal–Wallis analysis of biopsy +2 diagnostic modalities (non-mutually exclusive) with respect to (A) Time From Symptom Onset to Diagnosis, (B) Time From Treatment 1 to Progression, (C) Survival Time from Diagnosis, (D) Survival Time from Symptom Onset; Table S6: Kruskal–Wallis analysis of biopsy +3 diagnostic modalities (non-mutually exclusive) with respect to (A) Time From Symptom Onset to Diagnosis, (B) Time From Treatment 1 to Progression, (C) Survival Time from Diagnosis, (D) Survival Time from Symptom Onset; Table S7: Kruskal–Wallis analysis of biopsy +4 diagnostic modalities (non-mutually exclusive) with respect to (A) Time From Symptom Onset to Diagnosis, (B) Time From Treatment 1 to Progression, (C) Survival Time from Diagnosis, (D) Survival Time from Symptom Onset; Table S8: Kruskal–Wallis analysis of biopsy +2 diagnostic modalities (mutually exclusive) with respect to (A) Time From Symptom Onset to Diagnosis, (B) Time From Treatment 1 to Progression, (C) Survival Time from Diagnosis, (D) Survival Time from Symptom Onset; Table S9: Kruskal–Wallis analysis of biopsy +2 diagnostic modalities (mutually exclusive) with respect to (A) Time From Symptom Onset to Diagnosis, (B) Time From Treatment 1 to Progression, (C) Survival Time from Diagnosis, (D) Survival Time from Symptom Onset; Table S10: Kruskal–Wallis analysis of individual diagnostic modalities in patients without biopsy (non-mutually exclusive) with respect to (A) Time From Symptom Onset to Diagnosis, (B) Time From Treatment 1 to Progression, (C) Survival Time from Diagnosis, (D) Survival Time from Symptom Onset; Table S11: Kruskal–Wallis analysis of 2 diagnostic modalities in patients without biopsy (non-mutually exclusive) with respect to (A) Time From Symptom Onset to Diagnosis, (B) Time From Treatment 1 to Progression, (C) Survival Time from Diagnosis, (D) Survival Time from Symptom Onset; Table S12: Kruskal–Wallis analysis of 3 diagnostic modalities in patients without biopsy (non-mutually exclusive) with respect to (A) Time From Symptom Onset to Diagnosis, (B) Time From Treatment 1 to Progression, (C) Survival Time from Diagnosis, (D) Survival Time from Symptom Onset; Table S13: Kruskal–Wallis analysis of individual diagnostic modalities in patients without biopsy (mutually exclusive) with respect to (A) Time From Symptom Onset to Diagnosis, (B) Time From Treatment 1 to Progression, (C) Survival Time from Diagnosis, (D) Survival Time from Symptom Onset; Table S14: Kruskal-Wallis analysis of 2 diagnostic modalities in patients without biopsy (mutually exclusive) with respect to (A) Time From Symptom Onset to Diagnosis, (B) Time From Treatment 1 to Progression, (C) Survival Time from Diagnosis, (D) Survival Time from Symptom Onset; Table S15: Kruskal-Wallis analysis of 3 diagnostic modalities in patients without biopsy (mutually exclusive) with respect to (A) Time From Symptom Onset to Diagnosis, (B) Time From Treatment 1 to Progression, (C) Survival Time from Diagnosis, (D) Survival Time from Symptom Onset. Table S16: Citations of articles included in analysis (*n* = 231).
