# Supplementary material for: Diagnostic Considerations for Neurolymphomatosis: A Natural History Analysis
Source: Cancers (Basel). 2026 Jun 25;18(13):2068. doi: 10.3390/cancers18132068 (PMC13359875; doi:10.3390/cancers18132068)
Supplement: Supplementary file 1 [file cancers-18-02068-s001.zip › cancers-4346891-supplementary materials(Tables).pdf]

cancers

MDPI

Supplementary Materials: Diagnostic Considerations for Neurolymphomatosis: A Natural History Analysis

Francesca Rothell, Mary Ann Nguyen, Elizabeth Xu, Quan Ho, Sibo Zhou, Shiva Gautam and Eric T. Wong

Table S1. Kruskal-Wallis analysis of individual diagnostic modalities (non-mutually exclusive) with respect to (A) Time From Symptom Onset to Diagnosis, (B) Time From Treatment 1 to Progression, (C) Survival Time from Diagnosis, (D) Survival Time from Symptom Onset.:

1A.

Symptom Onset to Diagnosis						
**Diagnostic Method**	**N**	**Median**	**Mean**	**Standard Deviation**	**Lower Bound**	**Upper Bound**
Biopsy	72	3.75	11.50694444	19.67589932	7.364149306	16.21788194
CT	25	2	6.29	10.69750094	2.82975	11.76
EMG	17	5	16.58823529	23.6014298	5.941176471	28.06176471
MRI	86	3	8.877790698	16.66385703	5.528255814	12.49646802
PET	74	3	8.146216216	15.60084289	5.00039527	11.91804054
Ultrasound	8	4.5	9.875	10.62098852	3.621875	18.634375

Statistic	Degrees of Freedom	P Value	Method
7.097595179	5	0.2134826358	Kruskal-Wallis rank sum test

Comparison	Z Value	P Unadjusted	Bonferroni Adjusted P Value
Biopsy vs. CT	1.469838546	0.1416054868	1
Biopsy vs. EMG	-1.152320593	0.2491893577	1
CT vs. EMG	-2.073831582	0.03809495494	0.5714243242
Biopsy vs. MRI	0.9937335551	0.320352602	1
CT vs. MRI	-0.8030589865	0.4219406417	1
EMG vs. MRI	1.768715986	0.07694128312	1
Biopsy vs. PET	1.50339433	0.1327373879	1
CT vs. PET	-0.3991748985	0.6897643375	1
EMG vs. PET	2.080615708	0.037469095	0.562036425
MRI vs. PET	0.5684151937	0.569753076	1

Biopsy vs. Ultrasound	-0.22128978	0.8248668071	1
CT vs. Ultrasound	-1.043022801	0.2969377327	1
EMG vs. Ultrasound	0.5323803336	0.5944626033	1
MRI vs. Ultrasound	-0.6525639716	0.5140374188	1
PET vs. Ultrasound	-0.8902737714	0.3733189011	1

1B

Treatment 1 to Progression

Diagnostic Method	N	Median	Mean	Standard Deviation	Lower Bound	Upper Bound
Biopsy	67	16	20.24850746	13.91258251	17.05645522	23.42098881
CT	12	4.98	14.27416667	19.14365309	5.242875	27.6096875
EMG	3	6	5	1.152853018	3	6
MRI	45	6.4	6.954444444	4.489269426	5.659888889	8.324833333
PET	46	6.4	9.353043478	10.90551071	6.576141304	13.49505435

Statistic	Degrees of Freedom	P Value	Method
31.28778514	4	0.000002674229556	Kruskal-Wallis rank sum test

Comparison	Z Value	P Unadjusted	Bonferroni Adjusted P Value
Biopsy vs. CT	2.487960359	0.01284780528	0.1284780528
Biopsy vs. EMG	2.265629871	0.02347405461	0.2347405461
CT vs. EMG	0.8631240277	0.3880692785	1
Biopsy vs. MRI	4.751617622	0.000002017957165	0.00002017957165
CT vs. MRI	0.4183865593	0.6756645119	1
EMG vs. MRI	-0.7063960768	0.4799418599	1
Biopsy vs. PET	4.117114146	0.00003836460871	0.0003836460871
CT vs. PET	0.02610510107	0.9791735084	1
EMG vs. PET	-0.9207938188	0.3571580828	1
MRI vs. PET	-0.6079516076	0.54321957	1

1C

Survival Time from Diagnosis

Diagnostic Method	N	Median	Mean	Standard Deviation	Lower Bound	Upper Bound
Biopsy	234	10.5	28.14478632	39.90405962	23.19453098	33.20471474
CT	78	10	13.35871795	16.70452899	9.727948718	17.36953205
EMG	35	7	13.50942857	16.92527349	8.6041	19.9165
MRI	259	10	11.86378378	14.49942087	10.16848552	13.82120753
PET	222	8.02	12.39256757	16.05056749	10.44008333	14.7115
Ultrasound	10	4.5	8.75	8.135702015	4.04875	14.6

Statistic	Degrees of Freedom	P Value	Method
27.32365837	5	0.00004933219733	Kruskal-Wallis rank sum test

Comparison	Z Value	P Unadjusted	Bonferroni Adjusted P Value
Biopsy vs. CT	2.872824246	0.004068203696	0.06102305543
Biopsy vs. EMG	1.82451878	0.06807367983	1
CT vs. EMG	-0.2209098638	0.825162617	1
Biopsy vs. MRI	4.20220092	0.00002643323576	0.0003964985364
CT vs. MRI	0.02630943589	0.9790105288	1
EMG vs. MRI	0.2684333453	0.7883657752	1
Biopsy vs. PET	4.531164126	0.000005865954219	0.00008798931329
CT vs. PET	0.3716984486	0.7101173803	1
EMG vs. PET	0.5161370811	0.6057586709	1
MRI vs. PET	0.4977587836	0.6186540698	1
Biopsy vs. Ultrasound	1.858594172	0.06308468379	0.9462702569
CT vs. Ultrasound	0.6685611671	0.5037754511	1
EMG vs. Ultrasound	0.751615129	0.4522825425	1
MRI vs. Ultrasound	0.6862573119	0.4925508628	1
PET vs. Ultrasound	0.5433104044	0.5869161019	1

1D

Survival Time from Symptom Onset						
Diagnostic Method	N	Median	Mean	Standard Deviation	Lower Bound	Upper Bound

Biopsy	248	12	29.89669355	40.37070684	25.14962702	35.22022379
CT	82	10	14.6247561	18.49134884	10.73870427	18.97902744
EMG	37	13	20.40081081	24.15421487	13.04822297	28.52265541
MRI	276	10	13.89931159	17.53906592	11.93313496	16.13756069
PET	236	9.15	14.21173729	18.30796503	12.02587924	16.673625
Ultrasound	14	4.5	11.89285714	15.48433519	4.676785714	21.78660714

Statistic	Degrees of Freedom	P Value	Method
30.54003145	5	0.00001154424528	Kruskal-Wallis rank sum test

Comparison	Z Value	P Unadjusted	Bonferroni Adjusted P Value
Biopsy vs. CT	3.261621073	0.001107771148	0.01661656723
Biopsy vs. EMG	0.7289926223	0.4660061754	1
CT vs. EMG	-1.44922109	0.1472758497	1
Biopsy vs. MRI	4.236471664	0.00002270595604	0.0003405893406
CT vs. MRI	-0.3563260741	0.7215963848	1
EMG vs. MRI	1.383412014	0.1665385689	1
Biopsy vs. PET	4.592504231	0.000004379586295	0.00006569379443
CT vs. PET	0.01670587509	0.9866712602	1
EMG vs. PET	1.635325796	0.1019807687	1
MRI vs. PET	0.5296334625	0.5963660889	1
Biopsy vs. Ultrasound	2.39663097	0.01654657893	0.248198684
CT vs. Ultrasound	0.8398661722	0.4009834263	1
EMG vs. Ultrasound	1.688727281	0.09127170508	1
MRI vs. Ultrasound	1.050118212	0.2936637665	1
PET vs. Ultrasound	0.8751417554	0.38149678	1

Table S2. Kruskal-Wallis analysis of individual diagnostic modalities (mutually exclusive) with respect to (A) Time From Symptom Onset to Diagnosis, (B) Time From Treatment 1 to Progression, (C) Survival Time from Diagnosis, (D) Survival Time from Symptom Onset.

2A

Symptom Onset to Diagnosis

Diagnostic Method	N	Median	Mean	Standard Deviation	Lower Bound	Upper Bound
Biopsy	3	6	23	22.10210854	3	60
MRI	2	1.625	1.625	0.9847169035	0.25	3
PET	7	2	4.297142857	4.623722776	1.178571429	8.298035714

Statistic	Degrees of Freedom	P Value	Method
2.728638973	2	0.2555545251	Kruskal-Wallis rank sum test

Comparison	Z Value	P Unadjusted	Bonferroni Adjusted P Value
Biopsy vs. MRI	1.478850106	0.1391803808	0.4175411424
Biopsy vs. PET	1.40701912	0.1594217193	0.478265158
MRI vs. PET	-0.4727756655	0.636373237	1

2B

Treatment 1 to Progression

Diagnostic Method	N	Median	Mean	Standard Deviation	Lower Bound	Upper Bound
Biopsy	41	36	28.22926829	11.00180543	24.91646341	31.6
PET	1	2	2	NA	NA	NA

2C

Survival Time from Diagnosis

Diagnostic Method	N	Median	Mean	Standard Deviation	Lower Bound	Upper Bound
Biopsy	64	25.4	65.634375	55.31113673	52.54328125	78.81382813
CT	2	0	0	0	0	NA
EMG	1	0	0	NA	NA	NA
MRI	36	10	10.00638889	8.506745385	7.3395625	13.20846528
PET	24	5	9.637916667	13.44495494	4.825979167	16.18802083

Statistic	Degrees of Freedom	P-Value	Method
53.10704599	3	0.00000000001739506	Kruskal-Wallis rank sum test

Comparison	Z Value	P Unadjusted	Bonferroni Adjusted P Value
Biopsy vs. CT	3.061309974	0.002203708239	0.01322224943

Biopsy vs. MRI	5.652791219	0.00000001578630151	0.00000009471780905
CT vs. MRI	-1.404810309	0.1600776929	0.9604661574
Biopsy vs. PET	5.585142625	0.00000002335084982	0.0000001401050989
CT vs. PET	-1.170404146	0.2418383679	1
MRI vs. PET	0.6040338668	0.5458211262	1

2D

Survival Time from Symptom Onset						
**Diagnostic Method**	**N**	**Median**	**Mean**	**Standard Deviation**	**Lower Bound**	**Upper Bound**
Biopsy	64	25.4	66.7125	56.46751281	53.16820313	82.25164063
CT	2	0	0	0	0	0
EMG	1	0	0	NA	NA	NA
MRI	36	10	10.09666667	8.486168095	7.442625	13.11145833
PET	25	5	10.4556	13.64390346	5.6008	16.14373

**Statistic**	**Degrees of Freedom**	**P Value**	**Method**
53.69793165	3	0.0000000000130147	Kruskal-Wallis rank sum test

**Comparison**	**Z Value**	**P Unadjusted**	**Bonferroni Adjusted P Value**
Biopsy vs. CT	3.128495085	0.001757039493	0.01054223696
Biopsy vs. MRI	5.769476292	0.000000007951826391	0.00000004771095835
CT vs. MRI	-1.437755663	0.1505033933	0.9030203599
Biopsy vs. PET	5.520960917	0.00000003371507716	0.000000202290463
CT vs. PET	-1.285115911	0.1987517822	1
MRI vs. PET	0.3846553111	0.7004928089	1

Table S3. Kruskal-Wallis analysis of biopsy +1 diagnostic modalities (non-mutually exclusive) with respect to (A) Time From Symptom Onset to Diagnosis, (B) Time From Treatment 1 to Progression, (C) Survival Time from Diagnosis, (D) Survival Time from Symptom Onset.

3A

Symptom Onset to Diagnosis						
**Diagnostic Method**	**N**	**Median**	**Mean**	**Standard Deviation**	**Lower Bound**	**Upper Bound**
Biopsy + CT	23	3	6.793478261	11.18117513	2.891032609	12.74048913
Biopsy + EMG	15	5	17.06666667	24.61894056	5.598333333	31.14166667
Biopsy + MRI	56	4	11.91071429	19.88567966	7.048325893	17.85301339
Biopsy + PET	43	3	11.29069767	19.52202815	6	17.53575581

Biopsy + Ultrasound	7	6	10.85714286	11.15033421	3.428571429	20.29285714

Statistic	Degrees of Freedom	P Value	Method
3.48746982	4	0.479786142	Kruskal-Wallis rank sum test

Comparison	Z Value	P Unadjusted	Bonferroni Adjusted P Value
Biopsy + CT vs. Biopsy + EMG	-1.684658356	0.09205450551	0.9205450551
Biopsy + CT vs. Biopsy + MRI	-1.276338134	0.2018360154	1
Biopsy + EMG vs. Biopsy + MRI	0.8358558827	0.403235984	1
Biopsy + CT vs. Biopsy + PET	-0.6536079091	0.5133644474	1
Biopsy + EMG vs. Biopsy + PET	1.301426553	0.1931124898	1
Biopsy + MRI vs. Biopsy + PET	0.7262240329	0.4677014412	1
Biopsy + CT vs. Biopsy + Ultrasound	-0.8403748951	0.4006982206	1
Biopsy + EMG vs. Biopsy + Ultrasound	0.4289460444	0.6679624908	1
Biopsy + MRI vs. Biopsy + Ultrasound	-0.1163990303	0.9073363038	1
Biopsy + PET vs. Biopsy + Ultrasound	-0.4757838175	0.6342283998	1

3B

Treatment 1 to Progression						
**Diagnostic Method**	**N**	**Median**	**Mean**	**Standard Deviation**	**Lower Bound**	**Upper Bound**
Biopsy + CT	9	3	7.472222222	7.697598957	2.831944444	13.64097222
Biopsy + EMG	2	4.5	4.5	1.065751341	3	6
Biopsy + MRI	22	6	7.113636364	5.933197714	4.863068182	9.772727273
Biopsy + PET	19	7	8.828947368	7.423131111	5.63125	12.28980263

Statistic	Degrees of Freedom	P Value	Method
0.6333753733	3	0.8887511172	Kruskal-Wallis rank sum test

Comparison	Z Value	P Unadjusted	Bonferroni Adjusted P Value
Biopsy + CT vs. Biopsy + EMG	0.07778815228	0.937996571	1
Biopsy + CT vs. Biopsy + MRI	-0.3226068913	0.7469929741	1
Biopsy + EMG vs. Biopsy + MRI	-0.2551761969	0.7985870147	1
Biopsy + CT vs. Biopsy + PET	-0.6974579571	0.4855162418	1

Biopsy + EMG vs. Biopsy + PET	-0.4614484756	0.6444768795	1
Biopsy + MRI vs. Biopsy + PET	-0.4935611804	0.6216161221	1

3C

Survival Time from Diagnosis						
**Diagnostic Method**	**N**	**Median**	**Mean**	**Standard Deviation**	**Lower Bound**	**Upper Bound**
Biopsy + CT	51	10	11.60392157	8.58603693	9.390686275	14.04840686
Biopsy + EMG	24	10	16.47208333	19.355051	9.56053125	25.45523958
Biopsy + MRI	141	10	14.47184397	17.53180959	11.75500887	17.50955319
Biopsy + PET	106	10	14.40283019	16.0553566	11.44196934	17.73299528
Biopsy + Ultrasound	10	4.5	8.75	8.133656449	4.04875	14.30125

Statistic	Degrees of Freedom	P Value	Method
1.44713357	4	0.8359629968	Kruskal-Wallis rank sum test

Comparison	Z Value	P Unadjusted	Bonferroni Adjusted P Value
Biopsy + CT vs. Biopsy + EMG	-0.3021526833	0.7625356726	1
Biopsy + CT vs. Biopsy + MRI	-0.04114121349	0.9671833188	1
Biopsy + EMG vs. Biopsy + MRI	0.3082746997	0.7578733159	1
Biopsy + CT vs. Biopsy + PET	-0.1170779479	0.9067982846	1
Biopsy + EMG vs. Biopsy + PET	0.2426048449	0.8083115276	1
Biopsy + MRI vs. Biopsy + PET	-0.1029098646	0.9180345085	1
Biopsy + CT vs. Biopsy + Ultrasound	1.030177521	0.3029266799	1
Biopsy + EMG vs. Biopsy + Ultrasound	1.14529747	0.2520859535	1
Biopsy + MRI vs. Biopsy + Ultrasound	1.109254469	0.267320421	1
Biopsy + PET vs. Biopsy + Ultrasound	1.137313319	0.2554073289	1

3D

Survival Time from Symptom Onset						
**Diagnostic Method**	**N**	**Median**	**Mean**	**Standard Deviation**	**Lower Bound**	**Upper Bound**
Biopsy + CT	54	10	13.85277778	13.41476014	10.54418981	17.85652778
Biopsy + EMG	26	14.5	25.05115385	27.13368869	15.40583654	36.00096154
Biopsy + MRI	151	10	17.93066225	21.61168074	14.63560265	21.75675166
Biopsy + PET	114	10	17.65087719	20.32534864	14.1635307	21.46462719
Biopsy + Ultrasound	13	5	12.57692308	15.82301888	4.958653846	23.46730769

Statistic	Degrees of Freedom	P Value	Method
4.092481028	4	0.3936345642	Kruskal-Wallis rank sum test

Comparison	Z Value	P Unadjusted	Bonferroni Adjusted P Value
Biopsy + CT vs. Biopsy + EMG	-1.38204024	0.1669593459	1
Biopsy + CT vs. Biopsy + MRI	-0.5039582052	0.6142907473	1
Biopsy + EMG vs. Biopsy + MRI	1.177377436	0.2390448917	1
Biopsy + CT vs. Biopsy + PET	-0.3965517332	0.6916980515	1
Biopsy + EMG vs. Biopsy + PET	1.216523408	0.2237855995	1
Biopsy + MRI vs. Biopsy + PET	0.1160400743	0.9076207816	1
Biopsy + CT vs. Biopsy + Ultrasound	1.061508875	0.2884587019	1
Biopsy + EMG vs. Biopsy + Ultrasound	1.93662384	0.05279134158	0.5279134158
Biopsy + MRI vs. Biopsy + Ultrasound	1.411023365	0.1582377248	1
Biopsy + PET vs. Biopsy + Ultrasound	1.344032564	0.178937857	1

Table S4. Kruskal-Wallis analysis of biopsy +1 diagnostic modalities (mutually exclusive) with respect to (A) Time From Symptom Onset to Diagnosis, (B) Time From Treatment 1 to Progression, (C) Survival Time from Diagnosis, (D) Survival Time from Symptom Onset.

10A

Symptom Onset to Diagnosis						
**Diagnostic Method**	**N**	**Median**	**Mean**	**Standard Deviation**	**Lower Bound**	**Upper Bound**
Biopsy + MRI	11	4	13.29545455	22.85467617	2.840909091	30.84261364
Biopsy + PET	5	5	15	19.5547492	1.8	38

Statistic	P Value	Method
26.5	0.9547621856	Wilcoxon rank sum test with continuity correction

4B

Treatment 1 to Progression						
**Diagnostic Method**	**N**	**Median**	**Mean**	**Standard Deviation**	**Lower Bound**	**Upper Bound**
Biopsy + MRI	3	6	6.666666667	3.734446932	2	12

Biopsy + PET	2	7.5	7.5	3.141251165	3	12

Statistic	P Value	Method
2.5	1	Wilcoxon rank sum test with continuity correction

4C

Survival Time from Diagnosis

Diagnostic Method	N	Median	Mean	Standard Deviation	Lower Bound	Upper Bound
Biopsy + CT	4	9	8.5	5.392619384	2.5	14.0125
Biopsy + EMG	1	12	12	NA	NA	NA
Biopsy + MRI	30	6.5	16.05	26.12275421	7.21575	27.19475
Biopsy + PET	15	4	13.66666667	20.20035192	4.4	26.5

Statistic	Degrees of Freedom	P Value	Method
0.1207352865	2	0.9414183638	Kruskal-Wallis rank sum test

Comparison	Z Value	P Unadjusted	Bonferroni Adjusted P Value
Biopsy + CT vs. Biopsy + MRI	0.08145194523	0.93508254	1
Biopsy + CT vs. Biopsy + PET	0.2540429969	0.7994623407	1
Biopsy + MRI vs. Biopsy + PET	0.3149687724	0.752785364	1

4D

Survival Time from Symptom Onset

Diagnostic Method	N	Median	Mean	Standard Deviation	Lower Bound	Upper Bound
Biopsy + CT	4	9	8.5	5.335278445	2.5	14
Biopsy + EMG	1	12	12	NA	NA	NA
Biopsy + MRI	34	9	18.46323529	28.87495075	9.443419118	29.85974265
Biopsy + PET	16	6.25	17.5	23.22716587	7.02890625	31.31328125

Statistic	Degrees of Freedom	P Value	Method
0.04014818879	2	0.9801260488	Kruskal-Wallis rank sum test

Comparison	Z Value	P Unadjusted	Bonferroni Adjusted P Value
Biopsy + CT vs. Biopsy + MRI	-0.1578124831	0.8746045618	1
Biopsy + CT vs. Biopsy + PET	-0.06769572058	0.946027856	1
Biopsy + MRI vs. Biopsy + PET	0.1503307253	0.8805036931	1

Table S5. Kruskal-Wallis analysis of biopsy +2 diagnostic modalities (non-mutually exclusive) with respect to (A) Time From Symptom Onset to Diagnosis, (B) Time From Treatment 1 to Progression, (C) Survival Time from Diagnosis, (D) Survival Time from Symptom Onset.

5A

Symptom Onset to Diagnosis						
**Diagnostic Method**	**N**	**Median**	**Mean**	**Standard Deviation**	**Lower Bound**	**Upper Bound**
Biopsy + CT + EMG	2	5	5	2.095015971	2	8
Biopsy + CT + MRI	18	3.25	8.208333333	12.2182748	3.375	15.18125
Biopsy + CT + PET	14	2	6.607142857	12.36739653	1.785714286	15.32142857
Biopsy + CT + Ultrasound	1	1	1	NA	NA	NA
Biopsy + EMG + MRI	13	6	19.07692308	26.03929093	6.076923077	35.16153846
Biopsy + EMG + PET	10	5	19.8	28.31433122	4	40.8
Biopsy + EMG + Ultrasound	3	18	20	11.00226757	6	36
Biopsy + MRI + PET	30	3.25	13.1	20.7663875	6.595416667	21.85041667
Biopsy + MRI + Ultrasound	5	12	14.8	11.2146291	6	25.6
Biopsy + PET + Ultrasound	4	3.5	6.5	6.233549843	1	15

Statistic	Degrees of Freedom	P Value	Method
10.64641515	8	0.2225496307	Kruskal-Wallis rank sum test

Comparison	Z Value	P Unadjusted	Bonferroni Adjusted P Value
Biopsy + CT + EMG vs. Biopsy + CT + MRI	0.1071509596	0.9146692199	1
Biopsy + CT + EMG vs. Biopsy + CT + PET	0.621213266	0.5344593121	1
Biopsy + CT + MRI vs. Biopsy + CT + PET	1.093670027	0.2740997244	1
Biopsy + CT + EMG vs. Biopsy + EMG + MRI	-0.4536355293	0.6500911768	1
Biopsy + CT + MRI vs. Biopsy + EMG + MRI	-1.166083375	0.2435807375	1
Biopsy + CT + PET vs. Biopsy + EMG + MRI	-2.113782886	0.0345338153	1
Biopsy + CT + EMG vs. Biopsy + EMG + PET	-0.314599219	0.7530659722	1

Biopsy + CT + MRI vs. Biopsy + EMG + PET	-0.8203568806	0.412012689	1
Biopsy + CT + PET vs. Biopsy + EMG + PET	-1.722736309	0.08493622119	1
Biopsy + EMG + MRI vs. Biopsy + EMG + PET	0.2398194865	0.8104701997	1
Biopsy + CT + EMG vs. Biopsy + EMG + Ultrasound	-1.110675808	0.2667079189	1
Biopsy + CT + MRI vs Biopsy + EMG + Ultrasound	-1.753932796	0.07944202322	1
Biopsy + CT + PET vs. Biopsy + EMG + Ultrasound	-2.331775757	0.01971249103	0.7096496771
Biopsy + EMG + MRI vs. Biopsy + EMG + Ultrasound	-1.045011015	0.2960178788	1
Biopsy + EMG + PET vs. Biopsy + EMG + Ultrasound	-1.170042324	0.2419839369	1
Biopsy + CT + EMG vs. Biopsy + MRI + PET	-0.09122265284	0.9273156756	1
Biopsy + CT + MRI vs. Biopsy + MRI + PET	-0.4913263516	0.6231956449	1
Biopsy + CT + PET vs. Biopsy + MRI + PET	-1.656667679	0.09758668101	1
Biopsy + EMG + MRI vs. Biopsy + MRI + PET	0.8370495752	0.4025647024	1
Biopsy + EMG + PET vs. Biopsy + MRI + PET	0.4849204177	0.6277327984	1
Biopsy + EMG + Ultrasound vs. Biopsy + MRI + PET	1.564388123	0.1177264483	1
Biopsy + CT + EMG vs. Biopsy + MRI + Ultrasound	-0.7815887091	0.4344563245	1
Biopsy + CT + MRI vs. Biopsy + MRI + Ultrasound	-1.451539681	0.1466296412	1
Biopsy + CT + PET vs. Biopsy + MRI + Ultrasound	-2.156512173	0.03104369432	1
Biopsy + EMG + MRI vs. Biopsy + MRI + Ultrasound	-0.5878803609	0.5566125994	1

Biopsy + EMG + PET vs. Biopsy + MRI + Ultrasound	-0.7489859917	0.4538656486	1
Biopsy + EMG + Ultrasound vs. Biopsy + MRI + Ultrasound	0.4929224048	0.6220674158	1
Biopsy + MRI + PET vs. Biopsy + MRI + Ultrasound	-1.215835667	0.2240475288	1
Biopsy + CT + EMG vs. Biopsy + PET + Ultrasound	0.3289585652	0.7421870068	1
Biopsy + CT + MRI vs. Biopsy + PET + Ultrasound	0.3708966989	0.710714474	1
Biopsy + CT + PET vs. Biopsy + PET + Ultrasound	-0.3257918462	0.7445818483	1
Biopsy + EMG + MRI vs. Biopsy + PET + Ultrasound	1.100871556	0.2709525633	1
Biopsy + EMG + PET vs. Biopsy + PET + Ultrasound	0.8934530987	0.371614578	1
Biopsy + EMG + Ultrasound vs. Biopsy + PET + Ultrasound	1.700515454	0.08903401206	1
Biopsy + MRI + PET vs. Biopsy + PET + Ultrasound	0.6603649383	0.5090196666	1
Biopsy + MRI + Ultrasound vs. Biopsy + PET + Ultrasound	1.399496071	0.1616642759	1

5B

Treatment 1 to Progression

Diagnostic Method	N	Median	Mean	Standard Deviation	Lower Bound	Upper Bound
Biopsy + CT + EMG	1	3	3	NA	NA	NA
Biopsy + CT + MRI	7	3	5.642857143	4.467893402	2.5	9.430357143
Biopsy + CT + PET	5	12	11.15	8.732131945	3.9	19.2
Biopsy + EMG + MRI	2	4.5	4.5	1.070418245	3	6
Biopsy + EMG + PET	1	6	6	NA	NA	NA
Biopsy + MRI + PET	15	7	8.333333333	6.366317159	5.2	11.535

Statistic	Degrees of Freedom	P Value	Method
1.059953473	3	0.7867491901	Kruskal-Wallis rank sum test

Comparison	Z Value	P Unadjusted	Bonferroni Adjusted P Value

Biopsy + CT + MRI vs. Biopsy + CT + PET	-0.8445522647	0.3983608707	1
Biopsy + CT + MRI vs. Biopsy + EMG + MRI	0.0315754489	0.9748106226	1
Biopsy + CT + PET vs. Biopsy + EMG + MRI	0.6213226814	0.5343873332	1
Biopsy + CT + MRI vs. Biopsy + MRI + PET	-0.7878355888	0.4307928811	1
Biopsy + CT + PET vs. Biopsy + MRI + PET	0.2592906447	0.7954109971	1
Biopsy + EMG + MRI vs. Biopsy + MRI + PET	-0.5126896368	0.60816844	1

5C

Survival Time from Diagnosis

Diagnostic Method	N	Median	Mean	Standard Deviation	Lower Bound	Upper Bound
Biopsy + CT + EMG	4	7.5	6.875	5.150327124	1.5	12.253125
Biopsy + CT + MRI	43	10	12.16395349	8.467893338	9.670697674	14.71866279
Biopsy + CT + PET	26	10	11.98269231	6.955057667	9.309326923	14.80004808
Biopsy + EMG + MRI	20	10	16.4665	19.23040441	9.1405875	26.326875
Biopsy + EMG + PET	14	13.5	22.78571429	22.78139559	12.14107143	36.57142857
Biopsy + EMG + Ultrasound	3	10	12.5	7.563144802	3.5	19.45
Biopsy + MRI + PET	82	10	14.975	14.88450528	12.00972561	18.51847561
Biopsy + MRI + Ultrasound	8	7.5	10.3125	8.438121455	4.6875	16.6875
Biopsy + PET + Ultrasound	6	4	4	3.080363265	1.6625	6.504166667

Statistic	Degrees of Freedom	P Value	Method
10.09182315	8	0.2586402129	Kruskal-Wallis rank sum test

Comparison	Z Value	P Unadjusted	Bonferroni Adjusted P Value
Biopsy + CT + EMG vs. Biopsy + CT + MRI	-0.8908136853	0.373029132	1
Biopsy + CT + EMG vs. Biopsy + CT + PET	-0.952107188	0.341042624	1
Biopsy + CT + MRI vs. Biopsy + CT + PET	-0.183958408	0.8540460836	1
Biopsy + CT + EMG vs. Biopsy + EMG + MRI	-0.8575035593	0.3911666486	1
Biopsy + CT + MRI vs. Biopsy + EMG + MRI	-0.01482016907	0.9881756488	1
Biopsy + CT + PET vs. Biopsy + EMG + MRI	0.1401683395	0.8885269863	1

Biopsy + CT + EMG vs. Biopsy + EMG + PET	-1.457763334	0.144905788	1
Biopsy + CT + MRI vs. Biopsy + EMG + PET	-1.172575798	0.2409659593	1
Biopsy + CT + PET vs. Biopsy + EMG + PET	-0.9505689426	0.3418232407	1
Biopsy + EMG + MRI vs. Biopsy + EMG + PET	-1.023916411	0.3058747583	1
Biopsy + CT + EMG vs. Biopsy + EMG + Ultrasound	-0.5950213748	0.5518291832	1
Biopsy + CT + MRI vs. Biopsy + EMG + Ultrasound	0.01876872776	0.9850256011	1
Biopsy + CT + PET vs. Biopsy + EMG + Ultrasound	0.09333090962	0.9256406769	1
Biopsy + EMG + MRI vs. Biopsy + EMG + Ultrasound	0.02458083931	0.9803893027	1
Biopsy + EMG + PET vs. Biopsy + EMG + Ultrasound	0.5847434375	0.558720242	1
Biopsy + CT + EMG vs. Biopsy + MRI + PET	-1.029081408	0.3034414221	1
Biopsy + CT + MRI vs. Biopsy + MRI + PET	-0.3254559534	0.7448360137	1
Biopsy + CT + PET vs. Biopsy + MRI + PET	-0.06921153745	0.9448212396	1
Biopsy + EMG + MRI vs. Biopsy + MRI + PET	-0.2296288002	0.8183802255	1
Biopsy + EMG + PET vs. Biopsy + MRI + PET	1.035810019	0.3002907827	1
Biopsy + EMG + Ultrasound vs. Biopsy + MRI + PET	-0.1233139073	0.9018585285	1
Biopsy + CT + EMG vs. Biopsy + MRI + Ultrasound	-0.3503506379	0.7260755669	1
Biopsy + CT + MRI vs. Biopsy + MRI + Ultrasound	0.6521861616	0.514281086	1
Biopsy + CT + PET vs. Biopsy + MRI + Ultrasound	0.7341472868	0.4628589764	1
Biopsy + EMG + MRI vs. Biopsy + MRI + Ultrasound	0.6098748859	0.54194469	1
Biopsy + EMG + PET vs. Biopsy + MRI + Ultrasound	1.380698151	0.1673717902	1
Biopsy + EMG + Ultrasound vs. Biopsy + MRI + Ultrasound	0.3543705674	0.7230611891	1
Biopsy + MRI + PET vs. Biopsy + MRI + Ultrasound	0.8434048657	0.3990020516	1

Biopsy + CT + EMG vs. Biopsy + PET + Ultrasound	0.8961485933	0.370173407	1
Biopsy + CT + MRI vs. Biopsy + PET + Ultrasound	2.395874168	0.01658078139	0.5969081299
Biopsy + CT + PET vs. Biopsy + PET + Ultrasound	2.406268488	0.01611641535	0.5801909525
Biopsy + EMG + MRI vs. Biopsy + PET + Ultrasound	2.251756205	0.02433768267	0.8761565762
Biopsy + EMG + PET vs. Biopsy + PET + Ultrasound	2.879261092	0.003986081812	0.1434989452
Biopsy + EMG + Ultrasound vs. Biopsy + PET + Ultrasound	1.460764539	0.1440800706	1
Biopsy + MRI + PET vs. Biopsy + PET + Ultrasound	2.613735589	0.00895583158 8	0.3224099372
Biopsy + MRI + Ultrasound vs. Biopsy + PET + Ultrasound	1.46836272	0.1420057225	1

5D

Survival Time from Symptom Onset						
**Diagnostic Method**	**N**	**Median**	**Mean**	**Standard Deviation**	**Lower Bound**	**Upper Bound**
Biopsy + CT + EMG	4	8.5	9.375	7.84150392	1.5	17.25
Biopsy + CT + MRI	43	10	15.6	13.88008389	11.71738372	19.8405814
Biopsy + CT + PET	29	10	13.93275862	14.20017248	9.390387931	19.84637931
Biopsy + CT + Ultrasound	1	1	1	NA	NA	NA
Biopsy + EMG + MRI	22	15.5	26.24227273	27.99314043	14.75670455	38.70643182
Biopsy + EMG + PET	15	26	34.46666667	29.56546338	20.19666667	51.86833333
Biopsy + EMG + Ultrasound	4	17	24.375	19.18321811	6.625	49
Biopsy + MRI + PET	86	10	18.84825581	19.96374834	14.96148256	23.33828488
Biopsy + MRI + Ultrasound	10	8.5	15.65	16.99411595	5.9975	27.605
Biopsy + PET + Ultrasound	8	4.5	6.25	6.228699915	2.25	11.503125

**Statistic**	**Degrees of Freedom**	**P Value**	**Method**
16.09778593	8	0.04100168907	Kruskal-Wallis rank sum test

**Comparison**	**Z Value**	**P Unadjusted**	**Bonferroni Adjusted P Value**

Biopsy + CT + EMG vs. Biopsy + CT + MRI	-0.8664555731	0.3862403796	1
Biopsy + CT + EMG vs. Biopsy + CT + PET	-0.5071106444	0.6120771868	1
Biopsy + CT + MRI vs. Biopsy + CT + PET	0.7593067271	0.4476690943	1
Biopsy + CT + EMG vs. Biopsy + EMG + MRI	-1.257975182	0.2084007318	1
Biopsy + CT + MRI vs. Biopsy + EMG + MRI	-0.8806877253	0.3784868623	1
Biopsy + CT + PET vs. Biopsy + EMG + MRI	-1.461824565	0.1437892907	1
Biopsy + CT + EMG vs. Biopsy + EMG + PET	-1.982354795	0.04743954526	1
Biopsy + CT + MRI vs. Biopsy + EMG + PET	-2.209629952	0.02713085486	0.9767107748
Biopsy + CT + PET vs. Biopsy + EMG + PET	-2.657075209	0.00788218564	0.283758683
Biopsy + EMG + MRI vs. Biopsy + EMG + PET	-1.28940758	0.1972564275	1
Biopsy + CT + EMG vs. Biopsy + EMG + Ultrasound	-1.130960857	0.2580715667	1
Biopsy + CT + MRI vs. Biopsy + EMG + Ultrasound	-0.663390975	0.5070801845	1
Biopsy + CT + PET vs. Biopsy + EMG + Ultrasound	-0.9922448004	0.3210781231	1
Biopsy + EMG + MRI vs. Biopsy + EMG + Ultrasound	-0.2132774016	0.831110599	1
Biopsy + EMG + PET vs. Biopsy + EMG + Ultrasound	0.5612326692	0.5746389348	1
Biopsy + CT + EMG vs. Biopsy + MRI + PET	-0.9689408776	0.3325746885	1
Biopsy + CT + MRI vs. Biopsy + MRI + PET	-0.2285099873	0.8192497928	1
Biopsy + CT + PET vs. Biopsy + MRI + PET	-1.048422615	0.2944439385	1

Biopsy + EMG + MRI vs. Biopsy + MRI + PET	0.7875982494	0.430931739	1
Biopsy + EMG + PET vs. Biopsy + MRI + PET	2.215505161	0.02672541048	0.9621147773
Biopsy + EMG + Ultrasound vs. Biopsy + MRI + PET	0.5945326886	0.5521558855	1
Biopsy + CT + EMG vs. Biopsy + MRI + Ultrasound	-0.3905813181	0.6961067369	1
Biopsy + CT + MRI vs. Biopsy + MRI + Ultrasound	0.6319360469	0.5274286677	1
Biopsy + CT + PET vs. Biopsy + MRI + Ultrasound	0.1074561466	0.9144271138	1
Biopsy + EMG + MRI vs. Biopsy + MRI + Ultrasound	1.187017149	0.2352208564	1
Biopsy + EMG + PET vs. Biopsy + MRI + Ultrasound	2.166481411	0.03027441887	1
Biopsy + EMG + Ultrasound vs. Biopsy + MRI + Ultrasound	0.9611754547	0.3364639557	1
Biopsy + MRI + PET vs. Biopsy + MRI + Ultrasound	0.7917751626	0.4284917871	1
Biopsy + CT + EMG vs. Biopsy + PET + Ultrasound	0.6144567389	0.538913552	1
Biopsy + CT + MRI vs. Biopsy + PET + Ultrasound	2.15355872	0.0312747945	1
Biopsy + CT + PET vs. Biopsy + PET + Ultrasound	1.619503838	0.1053389013	1
Biopsy + EMG + MRI vs. Biopsy + PET + Ultrasound	2.567587914	0.01024088176	0.3686717432
Biopsy + EMG + PET vs. Biopsy + PET + Ultrasound	3.40753354	0.0006555285154	0.02359902655
Biopsy + EMG + Ultrasound vs. Biopsy + PET + Ultrasound	1.92037785	0.05481018932	1
Biopsy + MRI + PET vs. Biopsy + PET + Ultrasound	2.358792591	0.01833449989	0.6600419961

Biopsy + MRI + Ultrasound vs. Biopsy + PET + Ultrasound	1.28040073	0.2004042372	1

Table S6. Kruskal-Wallis analysis of biopsy +3 diagnostic modalities (non-mutually exclusive) with respect to (A) Time From Symptom Onset to Diagnosis, (B) Time From Treatment 1 to Progression, (C) Survival Time from Diagnosis, (D) Survival Time from Symptom Onset.

6A

Symptom Onset to Diagnosis						
**Diagnostic Method**	**N**	**Median**	**Mean**	**Standard Deviation**	**Lower Bound**	**Upper Bound**
Biopsy + CT + EMG + MRI	2	5	5	2.11368359	2	8
Biopsy + CT + EMG + PET	1	2	2	NA	NA	NA
Biopsy + CT + MRI + PET	9	2	9.333333333	14.89820158	2.166666667	22.22361111
Biopsy + CT + PET + Ultrasound	1	1	1	NA	NA	NA
Biopsy + EMG + MRI + PET	8	5.5	23.75	30.80511148	3.871875	46.628125
Biopsy + EMG + MRI + Ultrasound	3	18	20	11.20320249	6	36
Biopsy + EMG + PET + Ultrasound	2	12	12	4.218881899	6	18
Biopsy + MRI + PET + Ultrasound	2	12	12	4.239246575	6	18

**Statistic**	**Degrees of Freedom**	**P Value**	**Method**
5.099239453	5	0.4038894177	Kruskal-Wallis rank sum test

**Comparison**	**Z Value**	**P Unadjusted**	**Bonferroni Adjusted P Value**
Biopsy + CT + EMG + MRI vs. Biopsy + CT + MRI + PET	0.3155029908	0.7523797806	1
Biopsy + CT + EMG + MRI vs. Biopsy + EMG + MRI + PET	-0.3457364888	0.7295407593	1
Biopsy + CT + MRI + PET vs. Biopsy + EMG + MRI + PET	-1.070086812	0.2845802346	1
Biopsy + CT + EMG + MRI vs. Biopsy + EMG + MRI + Ultrasound	-1.100885212	0.270946619	1
Biopsy + CT + MRI + PET vs. Biopsy + EMG + MRI + Ultrasound	-1.877409209	0.06046203024	0.9069304535

Biopsy + EMG + MRI + PET vs. Biopsy + EMG + MRI + Ultrasound	-1.08070008	0.2798305477	1
Biopsy + CT + EMG + MRI vs. Biopsy + EMG + PET + Ultrasound	-0.7620072674	0.4460556667	1
Biopsy + CT + MRI + PET vs. Biopsy + EMG + PET + Ultrasound	-1.290265962	0.1969583306	1
Biopsy + EMG + MRI + PET vs. Biopsy + EMG + PET + Ultrasound	-0.6181349346	0.5364863957	1
Biopsy + EMG + MRI + Ultrasound vs. Biopsy + EMG + PET + Ultrasound	0.2661480732	0.7901251726	1
Biopsy + CT + EMG + MRI vs. Biopsy + MRI + PET + Ultrasound	-0.7620072674	0.4460556667	1
Biopsy + CT + MRI + PET vs. Biopsy + MRI + PET + Ultrasound	-1.290265962	0.1969583306	1
Biopsy + EMG + MRI + PET vs. Biopsy + MRI + PET + Ultrasound	-0.6181349346	0.5364863957	1
Biopsy + EMG + MRI + Ultrasound vs. Biopsy + MRI + PET + Ultrasound	0.2661480732	0.7901251726	1
Biopsy + EMG + PET + Ultrasound vs. Biopsy + MRI + PET + Ultrasound	0	1	1

6B

Treatment 1 to Progression						
**Diagnostic Method**	**N**	**Median**	**Mean**	**Standard Deviation**	**Lower Bound**	**Upper Bound**
Biopsy + CT + EMG + MRI	1	3	3	NA	NA	NA
Biopsy + CT + MRI + PET	3	12	9.333333333	3.86978616	3	13
Biopsy + EMG + MRI + PET	1	6	6	NA	NA	NA

6C

Survival Time from Diagnosis

Diagnostic Method	N	Median	Mean	Standard Deviation	Lower Bound	Upper Bound
Biopsy + CT + EMG + MRI	4	7.5	6.875	5.161325078	1.5	12.375
Biopsy + CT + EMG + PET	1	12	12	NA	NA	NA
Biopsy + CT + MRI + PET	22	10	12.58181818	5.811703649	10.40738636	14.93284091
Biopsy + EMG + MRI + PET	11	15	24.09090909	22.78038947	12.99318182	41.82045455
Biopsy + EMG + MRI + Ultrasound	3	10	12.5	7.611771218	3.5	24
Biopsy + EMG + PET + Ultrasound	1	10	10	NA	NA	NA
Biopsy + MRI + PET + Ultrasound	4	4.5	4.75	3.327325236	1.25	8.5

Statistic	Degrees of Freedom	P Value	Method
8.250194625	4	0.08283142174	Kruskal-Wallis rank sum test

Comparison	Z Value	P Unadjusted	Bonferroni Adjusted P Value
Biopsy + CT + EMG + MRI vs. Biopsy + CT + MRI + PET	-1.029481906	0.3032532779	1
Biopsy + CT + EMG + MRI vs. Biopsy + EMG + MRI + PET	-1.661013418	0.09671075182	0.9671075182
Biopsy + CT + MRI + PET vs. Biopsy + EMG + MRI + PET	-1.110936477	0.2665956928	1
Biopsy + CT + EMG + MRI vs. Biopsy + EMG + MRI + Ultrasound	-0.4923699688	0.6224578252	1
Biopsy + CT + MRI + PET vs. Biopsy + EMG + MRI + Ultrasound	0.2981987387	0.7655514844	1
Biopsy + EMG + MRI + PET vs. Biopsy + EMG + MRI + Ultrasound	0.9116131032	0.3619724222	1
Biopsy + CT + EMG + MRI vs. Biopsy + MRI + PET + Ultrasound	0.8257210686	0.4089623379	1
Biopsy + CT + MRI + PET vs. Biopsy + MRI + PET + Ultrasound	2.103651934	0.03540882015	0.3540882015

Biopsy + EMG + MRI + PET vs. Biopsy + MRI + PET + Ultrasound	2.661011293	0.0077906 3442	0.0779063442
Biopsy + EMG + MRI + Ultrasound vs. Biopsy + MRI + PET + Ultrasound	1.256839131	0.2088118 933	1

6D

Survival Time from Symptom Onset

Diagnostic Method	N	Median	Mean	Standard Deviation	Lower Bound	Upper Bound
Biopsy + CT + EMG + MRI	4	8.5	9.375	18.80993985	7.125	49
Biopsy + CT + EMG + PET	1	14	14	0.698904342 5	16	18
Biopsy + CT + MRI + PET	22	10	16.4	6.828473759	2.6	14.8
Biopsy + CT + PET + Ultrasound	1	1	1	7.80691341	1.5	17.25
Biopsy + EMG + MRI + PET	12	26.5	37.91666667	NA	NA	NA
Biopsy + EMG + MRI + Ultrasound	4	17	24.375	14.85248352	11.2725	24.26761 364
Biopsy + EMG + PET + Ultrasound	2	17	17	NA	NA	NA
Biopsy + MRI + PET + Ultrasound	5	5	8.6	29.94240067	21.1625	55.925

Statistic	Degrees of Freedom	P Value	Method
12.12640729	5	0.033096 16624	Kruskal-Wallis rank sum test

Comparison	Z Value	P Unadjusted	Bonferroni Adjusted P Value
Biopsy + EMG + MRI + Ultrasound vs. Biopsy + EMG + PET + Ultrasound	- 0.2240353745	0.822729 7779	1
Biopsy + EMG + MRI + Ultrasound vs. Biopsy + MRI + PET + Ultrasound	1.330450754	0.183369 8017	1
Biopsy + EMG + PET + Ultrasound vs. Biopsy + MRI + PET + Ultrasound	1.298632406	0.194070 1111	1
Biopsy + EMG + MRI + Ultrasound vs. Biopsy + CT + EMG + MRI	1.122488901	0.261654 6239	1
Biopsy + EMG + PET + Ultrasound	1.140543725	0.254059 8472	1

vs. Biopsy + CT + EMG + MRI			
Biopsy + MRI + PET + Ultrasound vs. Biopsy + CT + EMG + MRI	-0.1472435617	0.882939778	1
Biopsy + EMG + MRI + Ultrasound vs. Biopsy + CT + MRI + PET	0.7492905193	0.4536821208	1
Biopsy + EMG + PET + Ultrasound vs. Biopsy + CT + MRI + PET	0.8141676532	0.4155489133	1
Biopsy + MRI + PET + Ultrasound vs. Biopsy + CT + MRI + PET	-0.9793664965	0.3273989251	1
Biopsy + CT + EMG + MRI vs. Biopsy + CT + MRI + PET	-0.7109410045	0.4771207949	1
Biopsy + EMG + MRI + Ultrasound vs. Biopsy + EMG + MRI + PET	-0.9674254806	0.3333313772	1
Biopsy + EMG + PET + Ultrasound vs. Biopsy + EMG + MRI + PET	-0.4772726872	0.6331679574	1
Biopsy + MRI + PET + Ultrasound vs. Biopsy + EMG + MRI + PET	-2.726023586	0.006410241751	0.09615362626
Biopsy + CT + EMG + MRI vs. Biopsy + EMG + MRI + PET	-2.342188006	0.01917105507	0.287565826
Biopsy + CT + MRI + PET vs. Biopsy + EMG + MRI + PET	-2.691294734	0.007117528335	0.106762925

Table S7. Kruskal-Wallis analysis of biopsy +4 diagnostic modalities (non-mutually exclusive) with respect to (A) Time From Symptom Onset to Diagnosis, (B) Time From Treatment 1 to Progression, (C) Survival Time from Diagnosis, (D) Survival Time from Symptom Onset.

7A

**Symptom Onset to Diagnosis**						
**Diagnostic Method**	**N**	**Median**	**Mean**	**Standard Deviation**	**Lower Bound**	**Upper Bound**
Biopsy + CT + EMG + MRI + PET	1	2	2	NA	NA	NA
Biopsy + EMG + MRI + PET + Ultrasound	2	12	12	12	4.33258467	6

7B

**Treatment 1 to Progression**

NA

7C

Survival Time from Diagnosis						
Diagnostic Method	N	Median	Mean	Standard Deviation	Lower Bound	Upper Bound
Biopsy + CT + EMG + MRI + PET	1	12	12	NA	NA	NA
Biopsy + EMG + MRI + PET + Ultrasound	1	10	10	NA	NA	NA

7D

Survival Time from Symptom Onset						
Diagnostic Method	N	Median	Mean	Standard Deviation	Lower Bound	Upper Bound
Biopsy + CT + EMG + MRI + PET	1	14	14	NA	NA	NA
Biopsy + EMG + MRI + PET + Ultrasound	2	17	17	0.711	16	18

Table S8. Kruskal-Wallis analysis of biopsy +2 diagnostic modalities (mutually exclusive) with respect to (A) Time From Symptom Onset to Diagnosis, (B) Time From Treatment 1 to Progression, (C) Survival Time from Diagnosis, (D) Survival Time from Symptom Onset.

8A

Symptom Onset to Diagnosis						
Diagnostic Method	N	Median	Mean	Standard Deviation	Lower Bound	Upper Bound
Biopsy + CT + MRI	8	4	6.96875	6.558835701	2.71875	11.84609375
Biopsy + CT + PET	4	1.75	1.875	1.588038166	0.25	3.5
Biopsy + EMG + MRI	3	3	4.666666667	2.639257669	2	9
Biopsy + EMG + PET	2	4	4	0.7051268822	3	5
Biopsy + MRI + PET	14	4.5	8.642857143	8.959123446	4.428571429	13.14464286
Biopsy + MRI + Ultrasound	2	7	7	3.514320702	2	12
Biopsy + PET + Ultrasound	1	1	1	NA	NA	NA

Statistic	Degrees of Freedom	P Value	Method
2.909324594	5	0.7139629585	Kruskal-Wallis rank sum test

Comparison	Z Value	P Unadjusted	Bonferroni Adjusted P Value
Biopsy + CT + MRI vs. Biopsy + CT + PET	1.399826479	0.1615652866	1
Biopsy + CT + MRI vs. Biopsy + EMG + MRI	0.2174266843	0.8278758375	1

Biopsy + CT + PET vs. Biopsy + EMG + MRI	-0.9296299326	0.352562724	1
Biopsy + CT + MRI vs. Biopsy + EMG + PET	0	1	1
Biopsy + CT + PET vs. Biopsy + EMG + PET	-0.9898267955	0.3222587853	1
Biopsy + EMG + MRI vs. Biopsy + EMG + PET	-0.1612479447	0.8718981207	1
Biopsy + CT + MRI vs. Biopsy + MRI + PET	-0.1842036408	0.8538537034	1
Biopsy + CT + PET vs. Biopsy + MRI + PET	-1.655984015	0.097725056	1
Biopsy + EMG + MRI vs. Biopsy + MRI + PET	-0.3596901263	0.7190788766	1
Biopsy + EMG + PET vs. Biopsy + MRI + PET	-0.1079989575	0.9139965192	1
Biopsy + CT + MRI vs. Biopsy + MRI + Ultrasound	-0.09857281162	0.921477458	1
Biopsy + CT + PET vs. Biopsy + MRI + Ultrasound	-1.07981105	0.2802263297	1
Biopsy + EMG + MRI vs. Biopsy + MRI + Ultrasound	-0.2466145037	0.8052065788	1
Biopsy + EMG + PET vs. Biopsy + MRI + Ultrasound	-0.07792865002	0.9378848093	1
Biopsy + MRI + PET vs. Biopsy + MRI + Ultrasound	0.004909043523	0.9960831657	1

8B

Treatment 1 to Progression						
**Diagnostic Method**	**N**	**Median**	**Mean**	**Standard Deviation**	**Lower Bound**	**Upper Bound**
Biopsy + CT + MRI	3	1.5	2.833333333	1.907336674	1	6
Biopsy + CT + PET	2	13.875	13.875	9.32161192	0.75	27
Biopsy + MRI + PET	11	7	8.272727273	7.015948624	4.454545455	12.72954545

Statistic	Degrees of Freedom	P Value	Method
0.9308005427	2	0.627883731	Kruskal-Wallis rank sum test

Comparison	Z Value	P Unadjusted	Bonferroni Adjusted P Value

Biopsy + CT + MRI vs. Biopsy + CT + PET	-0.5408671865	0.5885991295	1
Biopsy + CT + MRI vs. Biopsy + MRI + PET	-0.9647800489	0.3346549909	1
Biopsy + CT + PET vs. Biopsy + MRI + PET	-0.1751734346	0.8609433577	1

8C

Survival Time from Diagnosis

Diagnostic Method	N	Median	Mean	Standard Deviation	Lower Bound	Upper Bound
Biopsy + CT + MRI	18	13.25	12.81944444	10.78292418	7.971180556	17.564375
Biopsy + CT + PET	4	3.875	8.6875	9.49635799	0.875	20.6875
Biopsy + EMG + MRI	4	5.165	5.3325	2.970220909	2.125	8.7075
Biopsy + EMG + PET	3	4	18	17.5347689	2	48
Biopsy + MRI + PET	47	10	14.66276596	14.19926479	11.04617021	18.73316489
Biopsy + MRI + Ultrasound	2	18	18	4.240943631	12	24
Biopsy + PET + Ultrasound	2	2.5	2.5	1.067872661	1	4

Statistic	Degrees of Freedom	P Value	Method
6.817753713	6	0.3380306943	Kruskal-Wallis rank sum test

Comparison	Z Value	P Unadjusted	Bonferroni Adjusted P Value
Biopsy + CT + MRI vs. Biopsy + CT + PET	0.8789864076	0.3794086457	1
Biopsy + CT + MRI vs. Biopsy + EMG + MRI	1.113093762	0.2656681627	1
Biopsy + CT + PET vs. Biopsy + EMG + MRI	0.1830101371	0.8547900647	1
Biopsy + CT + MRI vs. Biopsy + EMG + PET	0.1133641242	0.9097418811	1
Biopsy + CT + PET vs. Biopsy + EMG + PET	-0.5436022366	0.5867152203	1
Biopsy + EMG + MRI vs. Biopsy + EMG + PET	-0.7130367	0.4758230475	1

Biopsy + CT + MRI vs. Biopsy + MRI + PET	-0.4882921738	0.6253429041	1
Biopsy + CT + PET vs. Biopsy + MRI + PET	-1.192733305	0.2329738377	1
Biopsy + EMG + MRI vs. Biopsy + MRI + PET	-1.441191877	0.1495304825	1
Biopsy + EMG + PET vs. Biopsy + MRI + PET	-0.34600465	0.7293392202	1
Biopsy + CT + MRI vs. Biopsy + MRI + Ultrasound	-1.077078727	0.281445096	1
Biopsy + CT + PET vs. Biopsy + MRI + Ultrasound	-1.488045381	0.1367389262	1
Biopsy + EMG + MRI vs. Biopsy + MRI + Ultrasound	-1.637472532	0.1015317738	1
Biopsy + EMG + PET vs. Biopsy + MRI + Ultrasound	-0.9568735376	0.3386310892	1
Biopsy + MRI + PET vs. Biopsy + MRI + Ultrasound	-0.924465381	0.3552440678	1
Biopsy + CT + MRI vs. Biopsy + PET + Ultrasound	1.411455406	0.1581103758	1
Biopsy + CT + PET vs. Biopsy + PET + Ultrasound	0.6537437865	0.5132768882	1
Biopsy + EMG + MRI vs. Biopsy + PET + Ultrasound	0.5043166353	0.6140388894	1
Biopsy + EMG + PET vs. Biopsy + PET + Ultrasound	1.075006073	0.2823720093	1
Biopsy + MRI + PET vs. Biopsy + PET + Ultrasound	1.644588638	0.1000546716	1
Biopsy + MRI + Ultrasound vs.	1.854843828	0.06361853127	1

Biopsy + PET + Ultrasound

8D

Survival Time from Symptom Onset						
**Diagnostic Method**	**N**	**Median**	**Mean**	**Standard Deviation**	**Lower Bound**	**Upper Bound**
Biopsy + CT + MRI	18	17.75	15.91666667	12.32194852	10.30888889	21.58333333
Biopsy + CT + PET	6	3.5	7.041666667	7.945324022	1.833333333	15.08333333
Biopsy + EMG + MRI	5	5.5	7.066	3.379301983	4.232	9.9
Biopsy + EMG + PET	3	5	20.66666667	18.75834795	4	53
Biopsy + MRI + PET	50	10	16.203	15.41716763	12.1678	21.196325
Biopsy + MRI + Ultrasound	3	12	16.66666667	12.70865725	2	36
Biopsy + PET + Ultrasound	2	3	3	1.43061844	1	5

Statistic	Degrees of Freedom	P Value	Method
7.237709332	6	0.2994224592	Kruskal-Wallis rank sum test

Comparison	Z Value	P Unadjusted	Bonferroni Adjusted P Value
Biopsy + CT + MRI vs. Biopsy + CT + PET	1.770927319	0.07657278881	1
Biopsy + CT + MRI vs. Biopsy + EMG + MRI	1.172972048	0.2408070151	1
Biopsy + CT + PET vs. Biopsy + EMG + MRI	-0.3994127236	0.6895891203	1
Biopsy + CT + MRI vs. Biopsy + EMG + PET	0.1942697551	0.8459646664	1
Biopsy + CT + PET vs. Biopsy + EMG + PET	-1.009288393	0.3128363452	1
Biopsy + EMG + MRI vs. Biopsy + EMG + PET	-0.6460637489	0.5182380711	1
Biopsy + CT + MRI vs. Biopsy + MRI + PET	0.1118684156	0.91092774	1
Biopsy + CT + PET vs. Biopsy + MRI + PET	-1.861068422	0.06273451488	1

Biopsy + EMG + MRI vs. Biopsy + MRI + PET	-1.198650763	0.230663 7707	1
Biopsy + EMG + PET vs. Biopsy + MRI + PET	-0.1520793744	0.879124 3318	1
Biopsy + CT + MRI vs. Biopsy + MRI + Ultra- sound	- 0.01766088683	0.985909 3836	1
Biopsy + CT + PET vs. Biopsy + MRI + Ultra- sound	-1.196193651	0.231620 9981	1
Biopsy + EMG + MRI vs. Biopsy + MRI + Ultra- sound	-0.8270339867	0.408217 7982	1
Biopsy + EMG + PET vs. Biopsy + MRI + Ultra- sound	-0.1618647014	0.871412 4004	1
Biopsy + MRI + PET vs. Biopsy + MRI + Ultra- sound	- 0.07025889229	0.943987 6008	1
Biopsy + CT + MRI vs. Biopsy + PET + Ultra- sound	1.625377384	0.104082 1717	1
Biopsy + CT + PET vs. Biopsy + PET + Ultra- sound	0.4613143991	0.644573 0558	1
Biopsy + EMG + MRI vs. Biopsy + PET + Ultra- sound	0.7392704621	0.459742 7818	1
Biopsy + EMG + PET vs. Biopsy + PET + Ultra- sound	1.194403569	0.232320 1413	1
Biopsy + MRI + PET vs. Biopsy + PET + Ultra- sound	1.637384962	0.101550 0584	1
Biopsy + MRI + Ultrasound vs. Biopsy + PET + Ultrasound	1.33917976	0.180512 1599	1

Table S9: Kruskal-Wallis analysis of biopsy +3 diagnostic modalities (mutually exclusive) with respect to (A) Time From Symptom Onset to Diagnosis, (B) Time From Treatment 1 to Progression, (C) Survival Time from Diagnosis, (D) Survival Time from Symptom Onset.

9A

Symptom Onset to Diagnosis						
**Diagnostic Method**	**N**	**Median**	**Mean**	**Standard Deviation**	**Lower Bound**	**Upper Bound**
Biopsy + CT + EMG + MRI	1	8	8	NA	NA	NA
Biopsy + CT + MRI + PET	8	2.5	10.25	15.76349189	2.125	24.5640625
Biopsy + CT + PET + Ultrasound	1	1	1	NA	NA	NA
Biopsy + EMG + MRI + PET	5	5	32.8	36.38837629	2.8	70.015
Biopsy + EMG + MRI + Ultrasound	1	36	36	NA	NA	NA
**Statistic**	**P Value**	**Method**				
16	0.6028760629	Wilcoxon rank sum test with continuity correction				

9B

Treatment 1 to Progression						
**Diagnostic Method**	**N**	**Median**	**Mean**	**Standard Deviation**	**Lower Bound**	**Upper Bound**
Biopsy + CT + EMG + MRI	1	3	3	NA	NA	NA
Biopsy + CT + MRI + PET	3	12	9.333333333	3.77751649	3	13
Biopsy + EMG + MRI + PET	1	6	6	NA	NA	NA

9C

Survival Time from Diagnosis						
**Diagnostic Method**	**N**	**Median**	**Mean**	**Standard Deviation**	**Lower Bound**	**Upper Bound**
Biopsy + CT + EMG + MRI	3	3	5.166666667	4.675175819	0	9.333333333
Biopsy + CT + MRI + PET	21	10	12.60952381	5.969023075	10.21345238	15.38630952
Biopsy + EMG + MRI + PET	9	21	27	24.69943241	12.55277778	49.225
Biopsy + EMG + MRI + Ultrasound	2	13.75	13.75	7.27103761	3.5	24
Biopsy + MRI + PET + Ultrasound	3	4	3	1.862452356	0	5
**Statistic**	**Degrees of Freedom**	**P Value**	**Method**			
9.993695751	4	0.04053401024	Kruskal-Wallis rank sum test			
**Comparison**	**Z Value**	**P Unadjusted**	**Bonferroni Adjusted P Value**			

Biopsy + CT + EMG + MRI vs. Biopsy + CT + MRI + PET	-1.504230252	0.1325220909	1			
Biopsy + CT + EMG + MRI vs. Biopsy + EMG + MRI + PET	-2.095477597	0.03612855791	0.3612855791			
Biopsy + CT + MRI + PET vs. Biopsy + EMG + MRI + PET	-1.176061198	0.2395704218	1			
Biopsy + CT + EMG + MRI vs. Biopsy + EMG + MRI + Ultrasound	-0.856646782	0.3916401229	1			
Biopsy + CT + MRI + PET vs. Biopsy + EMG + MRI + Ultrasound	0.1978656453	0.8431501838	1			
Biopsy + EMG + MRI + PET - Biopsy + EMG + MRI + Ultrasound	0.7866813763	0.4314684089	1			
Biopsy + CT + EMG + MRI vs. Biopsy + MRI + PET + Ultrasound	0.5393212882	0.5896651823	1			
Biopsy + CT + MRI + PET vs. Biopsy + MRI + PET + Ultrasound	2.217685254	0.02657630035	0.2657630035			
Biopsy + EMG + MRI + PET vs. Biopsy + MRI + PET + Ultrasound	2.756008579	0.005851146232	0.05851146232			
Biopsy + EMG + MRI + Ultrasound vs. Biopsy + MRI + PET + Ultrasound	1.339030407	0.1805607743	1			

9D

Survival Time from Symptom Onset						
**Diagnostic Method**	**N**	**Median**	**Mean**	**Standard Deviation**	**Lower Bound**	**Upper Bound**
Biopsy + CT + EMG + MRI	3	3	7.833333333	7.749350438	0	20.5
Biopsy + CT + MRI + PET	21	10	16.51428571	14.99975435	11.0475	24.8477381
Biopsy + CT + PET + Ultrasound	1	1	1	NA	NA	NA
Biopsy + EMG + MRI + PET	9	30	45.22222222	31.6848871	24.775	66.79722222
Biopsy + EMG + MRI + Ultrasound	2	31.75	31.75	20.20748546	3.5	60
Biopsy + MRI + PET + Ultrasound	3	4	3	1.866306257	0	5
**Statistic**	**Degrees of Freedom**	**P Value**	**Method**			
14.72056927	4	0.005317286507	Kruskal-Wallis rank sum test			
**Comparison**	**Z Value**	**P Unadjusted**	**Bonferroni Adjusted P Value**			

Biopsy + CT + EMG + MRI vs. Biopsy + CT + MRI + PET	-1.24525654	0.2130374655	1			
Biopsy + CT + EMG + MRI vs. Biopsy + EMG + MRI + PET	-2.590994155	0.009569911683	0.09569911683			
Biopsy + CT + MRI + PET vs. Biopsy + EMG + MRI + PET	-2.406419341	0.016109761	0.16109761			
Biopsy + CT + EMG + MRI vs. Biopsy + EMG + MRI + Ultrasound	-0.9182709057	0.3584770551	1			
Biopsy + CT + MRI + PET vs. Biopsy + EMG + MRI + Ultrasound	-0.09415213606	0.9249883057	1			
Biopsy + EMG + MRI + PET vs. Biopsy + EMG + MRI + Ultrasound	1.137297844	0.2554137957	1			
Biopsy + CT + EMG + MRI vs. Biopsy + MRI + PET + Ultrasound	0.6159948501	0.5378979187	1			
Biopsy + CT + MRI + PET vs. Biopsy + MRI + PET + Ultrasound	2.060141131	0.0393850508	0.393850508			
Biopsy + EMG + MRI + PET vs. Biopsy + MRI + PET + Ultrasound	3.345430689	0.0008215488982	0.008215488982			
Biopsy + EMG + MRI + Ultrasound vs. Biopsy + MRI + PET + Ultrasound	1.469233449	0.1417694806	1			

Table S10: Kruskal-Wallis analysis of individual diagnostic modalities in patients without biopsy (non-mutually exclusive) with respect to (A) Time From Symptom Onset to Diagnosis, (B) Time From Treatment 1 to Progression, (C) Survival Time from Diagnosis, (D) Survival Time from Symptom Onset.

10A

Symptom Onset to Diagnosis						
**Diagnostic Method**	**N**	**Median**	**Mean**	**Standard Deviation**	**Lower Bound**	**Upper Bound**
CT	2	0.5	0.5	0.3532705479	0	1
EMG	2	13	13	7.818621101	2	24
MRI	30	2.065	3.216333333	4.044619257	1.955525	4.995675
PET	31	2.13	3.784516129	4.639509119	2.315435484	5.880201613
Ultrasound	1	3	3	NA	NA	NA

**Statistic**	**Degrees of Free-dom**	**P Value**	**Method**			
4.003670349	3	0.2610680712	Kruskal-Wallis rank sum test			
**Comparison**	**Z Value**	**P Unadjusted**	**Bonferroni Adjusted P Value**			
CT - EMG	-1.911039125	0.05599955316	0.3359973189			
CT - MRI	-1.547060724	0.1218486036	0.7310916215			
EMG - MRI	1.069737368	0.284737541	1			
CT - PET	-1.688787699	0.09126012196	0.5475607318			
EMG - PET	0.9306522901	0.3520334565	1			
MRI - PET	-0.3992799463	0.6896869417	1			

10B

**Treatment 1 to Progression**						
**Diagnostic Method**	**N**	**Median**	**Mean**	**Standard Devia-tion**	**Lower Bound**	**Upper Bound**
CT	3	21.24	34.68	28.15148838	3.96	78.84
EMG	1	6	6	NA	NA	NA
MRI	23	6.4	6.802173913	2.403851572	5.806467391	7.765217391
PET	27	6.4	9.721851852	12.38657803	5.984777778	15.55677778
**Statistic**	**Degrees of Free-dom**	**P Value**	**Method**			
1.511727634	2	0.4696047869	Kruskal-Wallis rank sum test			
**Comparison**	**Z Value**	**P Unadjusted**	**Bonferroni Adjusted P Value**			
CT - MRI	1.21244277	0.2253429371	0.6760288112			
CT - PET	1.174040573	0.2403787746	0.7211363237			
MRI - PET	-0.1048782432	0.9164724232	1			

10C

**Survival Time from Diagnosis**						
**Diagnostic Method**	**N**	**Median**	**Mean**	**Standard Devia-tion**	**Lower Bound**	**Upper Bound**
CT	27	3.84	16.67333333	25.46367983	7.732925926	27.94775926
EMG	11	6	7.045454545	4.486191406	4.636363636	9.682954545
MRI	118	9.4	8.747372881	8.320000756	7.324987288	10.37030085

PET	116	5.88	10.55560345	15.58386037	7.886299569	13.99927586
**Statistic**	**Degrees of Freedom**	**P Value**	**Method**			
1.123699965	3	0.771356117	Kruskal-Wallis rank sum test			
**Comparison**	**Z Value**	**P Unadjusted**	**Bonferroni Adjusted P Value**			
CT vs. EMG	-0.2453023959	0.8062222968	1			
CT vs. MRI	-0.9182490344	0.3584885028	1			
EMG vs. MRI	-0.3430611398	0.731552465	1			
CT vs. PET	-0.4484487397	0.6538293729	1			
EMG vs. PET	-0.02560977269	0.9795685911	1			
MRI vs. PET	0.7653692929	0.4440516727	1			

10D

Survival Time from Symptom Onset						
**Diagnostic Method**	**N**	**Median**	**Mean**	**Standard Deviation**	**Lower Bound**	**Upper Bound**
CT	28	3.72	16.11357143	25.13102928	7.481821429	25.33717857
EMG	11	6	9.409090909	7.775323428	5.271590909	14.41136364
MRI	125	9.4	9.02944	8.437232138	7.63574	10.76822
PET	122	6	10.99811475	15.35151525	8.39702459	14.06276639
Ultrasound	1	3	3	NA	NA	NA
**Statistic**	**Degrees of Freedom**	**P Value**	**Method**			
1.941302292	3	0.584678564	Kruskal-Wallis rank sum test			
**Comparison**	**Z Value**	**P Unadjusted**	**Bonferroni Adjusted P Value**			
CT - EMG	-0.8954598396	0.3705413249	1			
CT - MRI	-1.347088061	0.1779518728	1			
EMG - MRI	0.117626681	0.9063634633	1			
CT - PET	-1.00378084	0.3154842615	1			
EMG - PET	0.3440169039	0.7308335736	1			
MRI - PET	0.5602962043	0.575277416	1			

Table S11: Kruskal-Wallis analysis of 2 diagnostic modalities in patients without biopsy (non-mutually exclusive) with respect to (A) Time From Symptom Onset to Diagnosis, (B) Time From Treatment 1 to Progression, (C) Survival Time from Diagnosis, (D) Survival Time from Symptom Onset.

11A

Symptom Onset to Diagnosis						
**Diagnostic Method**	**N**	**Median**	**Mean**	**Standard Deviation**	**Lower Bound**	**Upper Bound**
CT + MRI	2	0.5	0.5	0.3579374526	0	1
EMG + MRI	2	13	13	7.691059038	2	24
EMG + PET	1	24	24	NA	NA	NA
MRI + PET	24	2.15	3.635	4.424493831	2.093291667	5.915572917
MRI + Ultrasound	1	3	3	NA	NA	NA
**Statistic**	**Degrees of Freedom**	**P Value**	**Method**			
3.698572902	2	0.1573494027	Kruskal-Wallis rank sum test			
**Comparison**	**Z Value**	**P Unadjusted**	**Bonferroni Adjusted P Value**			
CT + MRI vs. EMG + MRI	-1.858722328	0.06306650689	0.1891995207			
CT + MRI vs. MRI + PET	-1.625018415	0.1041586351	0.3124759054			
EMG + MRI vs. MRI + PET	0.9004879113	0.3678606557	1			

11B

Treatment 1 to Progression						
**Diagnostic Method**	**N**	**Median**	**Mean**	**Standard Deviation**	**Lower Bound**	**Upper Bound**
CT + PET	3	21.24	34.68	27.76794217	3.96	78.84
EMG + MRI	1	6	6	NA	NA	NA
EMG + PET	1	6	6	NA	NA	NA
MRI + PET	23	6.4	6.802173913	2.396160685	5.90826087	7.783043478
**Statistic**	**P Value**	**Method**				
49	0.2400213363	Wilcoxon rank sum test with continuity correction				

11C

Survival Time from Diagnosis						
**Diagnostic Method**	**N**	**Median**	**Mean**	**Standard Deviation**	**Lower Bound**	**Upper Bound**
CT + MRI	8	5.5	7.9375	8.350762658	2.6859375	14.4421875
CT + PET	19	4.2	20.87789474	28.88295349	9.033157895	35.07584211
EMG + MRI	9	6	8.055555556	4.230443414	5.555555556	10.83333333
EMG + PET	8	6	7.5625	4.224179023	4.75	10.8125
MRI + PET	74	6	8.127837838	7.910841901	6.57527027	10.19083446
**Statistic**	**Degrees of Freedom**	**P Value**	**Method**			
0.7901250351	4	0.9397670178	Kruskal-Wallis rank sum test			
**Comparison**	**Z Value**	**P Unadjusted**	**Bonferroni Adjusted P Value**			
CT + MRI vs. CT + PET	-0.6314091895	0.527773009	1			
CT + MRI vs. EMG + MRI	-0.831576498	0.4056480324	1			
CT + PET vs. EMG + MRI	-0.3409294377	0.7331567029	1			
CT + MRI vs. EMG + PET	-0.665867675	0.5054956836	1			
CT + PET vs. EMG + PET	-0.1585376185	0.8740331802	1			
EMG + MRI vs. EMG + PET	0.146404313	0.8836022227	1			
CT + MRI vs. MRI + PET	-0.6222367224	0.5337862225	1			
CT + PET vs. MRI + PET	0.1342823035	0.8931793462	1			
EMG + MRI vs. MRI + PET	0.4886186412	0.6251117131	1			
EMG + PET vs. MRI + PET	0.2723281983	0.7853696762	1			

11D

Survival Time from Symptom Onset						

Diagnostic Method	N	Median	Mean	Standard Deviation	Lower Bound	Upper Bound
CT + MRI	9	3	7.166666667	8.070210391	2.388888889	13.22222222
CT + PET	19	4.2	20.87789474	28.7916931	9.250473684	33.74589474
EMG + MRI	9	9	10.94444444	7.717372828	6.222222222	16.83333333
EMG + PET	8	7.5	10.5625	8.030981095	5.75	16.19375
MRI + PET	79	6	8.717721519	8.21896132	7.07585443	10.9435443
MRI + Ultrasound	1	3	3	NA	NA	NA

Statistic	Degrees of Freedom	P Value	Method
2.946552493	4	0.566809189	Kruskal-Wallis rank sum test

Comparison	Z-Value	P Unadjusted	Bonferroni Adjusted P Value
CT + MRI vs. CT + PET	-0.9600862394	0.3370118134	1
CT + MRI vs. EMG + MRI	-1.576031808	0.1150184729	1
CT + PET vs. EMG + MRI	-0.8759352542	0.3810652328	1
CT + MRI vs. EMG + PET	-1.326306559	0.1847381752	1
CT + PET vs. EMG + PET	-0.6073335225	0.5436295957	1
EMG + MRI vs. EMG + PET	0.2026688798	0.8393938487	1
CT + MRI vs. MRI + PET	-1.228094654	0.2194114376	1
CT + PET vs. MRI + PET	-0.1704539869	0.8646531179	1
EMG + MRI vs. MRI + PET	0.8837024294	0.3768568686	1
EMG + PET vs. MRI + PET	0.5725108044	0.5669759682	1

Table S12: Kruskal-Wallis analysis of 3 diagnostic modalities in patients without biopsy (non-mutually exclusive) with respect to (A) Time From Symptom Onset to Diagnosis, (B) Time From Treatment 1 to Progression, (C) Survival Time from Diagnosis, (D) Survival Time from Symptom Onset.

12A

Symptom Onset to Diagnosis						
Diagnostic Method	N	Median	Mean	Standard Deviation	Lower Bound	Upper Bound
EMG + MRI + PET	1	24	24	NA	NA	NA

12B

Treatment 1 to Progression						
Diagnostic Method	N	Median	Mean	Standard Deviation	Lower Bound	Upper Bound
EMG + MRI + PET	1	6	6	NA	NA	NA

12C

Survival Time from Diagnosis						
Diagnostic Method	N	Median	Mean	Standard Deviation	Lower Bound	Upper Bound
CT + MRI + PET	2	5	5	2.799011483	1	9
EMG + MRI + PET	7	6	7.928571429	4.37299152	4.714285714	11.71428571
Statistic	P Value	Method				
49	0.2400213363	Wilcoxon rank sum test with continuity correction				

12D

Survival Time from Symptom Onset						
Diagnostic Method	N	Median	Mean	Standard Deviation	Lower Bound	Upper Bound
CT + MRI + PET	2	5	5	2.831821237	1	9
EMG + MRI + PET	7	9	11.35714286	8.371167534	6.069642857	18.35892857
Statistic	P Value	Method				
3.5	0.3777807422	Wilcoxon rank sum test with continuity correction				

Table S13: Kruskal-Wallis analysis of individual diagnostic modalities in patients without biopsy (mutually exclusive) with respect to (A) Time From Symptom Onset to Diagnosis, (B) Time From Treatment 1 to Progression, (C) Survival Time from Diagnosis, (D) Survival Time from Symptom Onset.

13A

Symptom Onset to Diagnosis						
Diagnostic Method	N	Median	Mean	Standard Deviation	Lower Bound	Upper Bound

MRI	2	1.625	1.625	0.960993471	0.25	3
PET	7	2	4.297142857	4.594896608	1.178571429	8.106035714
**Statistic**	**P Value**	**Method**				
5.5	0.7687597568	Wilcoxon rank sum test with continuity correction				

13B

Treatment 1 to Progression						
**Diagnostic Method**	**N**	**Median**	**Mean**	**Standard Deviation**	**Lower Bound**	**Upper Bound**
PET	1	2	2	NA	NA	NA

13C

Survival Time from Diagnosis						
**Diagnostic Method**	**N**	**Median**	**Mean**	**Standard Deviation**	**Lower Bound**	**Upper Bound**
CT	2	0	0	0	0	0
EMG	1	0	0	NA	NA	NA
MRI	36	10	10.00638889	8.556240448	7.491388889	13.11145833
PET	24	5	9.637916667	13.33615429	5.05346875	16.90646875
**Statistic**	**Degrees of Freedom**	**P Value**	**Method**			
6.736755706	2	0.03444546766	Kruskal-Wallis rank sum test			
**Comparison**	**Z Value**	**P Unadjusted**	**Bonferroni Adjusted P Value**			
CT vs. MRI	-2.379632054	0.01732993247	0.05198979741			
CT vs. PET	-1.858137501	0.0631494906	0.1894484718			
MRI vs. PET	1.370697228	0.1704693592	0.5114080777			

13D

Survival Time from Symptom Onset						
**Diagnostic Method**	**N**	**Median**	**Mean**	**Standard Deviation**	**Lower Bound**	**Upper Bound**
CT	2	0	0	0	0	0
EMG	1	0	0	NA	NA	NA
MRI	36	10	10.09666667	8.521134614	7.499416667	13.13759722
PET	25	5	10.4556	13.65284363	5.76423	16.61562
**Statistic**	**Degrees of Freedom**	**P Value**	**Method**			
6.16857044	2	0.04576273251	Kruskal-Wallis rank sum test			

Comparison	Z Value	P Unadjusted	Bonferroni Adjusted P Value			
CT vs. MRI	-2.377412949	0.01743455764	0.05230367292			
CT vs. PET	-1.974803789	0.04829041888	0.1448712566			
MRI vs. PET	1.060037728	0.2891274358	0.8673823073			

Table S14: Kruskal-Wallis analysis of 2 diagnostic modalities in patients without biopsy (mutually exclusive) with respect to (A) Time From Symptom Onset to Diagnosis, (B) Time From Treatment 1 to Progression, (C) Survival Time from Diagnosis, (D) Survival Time from Symptom Onset.

14A

Symptom Onset to Diagnosis						
**Diagnostic Method**	**N**	**Median**	**Mean**	**Standard Deviation**	**Lower Bound**	**Upper Bound**
CT + MRI	2	0.5	0.5	0.3551090255	0	1
EMG + MRI	1	2	2	NA	NA	NA
MRI + PET	23	2.13	2.749565217	2.226322521	1.915152174	3.696630435
MRI + Ultrasound	1	3	3	NA	NA	NA
**Statistic**	**P Value**	**Method**				
7	0.1194496159	Wilcoxon rank sum test with continuity correction				

14B

Treatment 1 to Progression						
**Diagnostic Method**	**N**	**Median**	**Mean**	**Standard Deviation**	**Lower Bound**	**Upper Bound**
CT + PET	3	21.24	34.68	3.96	78.84	78.84
MRI + PET	22	6.4	6.838636364	5.824431818	7.790909091	7.830397727
**Statistic**	**P Value**	**Method**				
47	0.2351463464	Wilcoxon rank sum test with continuity correction				

14C

Survival Time from Diagnosis						
**Diagnostic Method**	**N**	**Median**	**Mean**	**Standard Deviation**	**Lower Bound**	**Upper Bound**

CT + MRI	6	5.5	8.916666667	9.17849694	2	17.41666667
CT + PET	17	4.2	22.74588235	29.96209233	9.136	39.08805882
EMG + MRI	2	8.5	8.5	2.439235552	5	12
EMG + PET	1	5	5	NA	NA	NA
MRI + PET	65	6	8.245538462	8.29347101	6.337769231	10.60007692
**Statistic**	**Degrees of Freedom**	**P Value**	**Method**			
0.4983272729	3	0.9192587485	Kruskal-Wallis rank sum test			
**Comparison**	**Z Value**	**P Unadjusted**	**Bonferroni Adjusted P Value**			
CT + MRI vs. CT + PET	-0.5520207304	0.5809341503	1			
CT + MRI vs. EMG + MRI	-0.4460098131	0.6555901575	1			
CT + PET vs. EMG + MRI	-0.1364920949	0.8914322734	1			
CT + MRI vs. MRI + PET	-0.2795461286	0.7798257429	1			
CT + PET vs. MRI + PET	0.5244130088	0.5999913105	1			
EMG + MRI vs. MRI + PET	0.3411188655	0.7330140995	1			

14D

Survival Time from Symptom Onset						
**Diagnostic Method**	**N**	**Median**	**Mean**	**Standard Deviation**	**Lower Bound**	**Upper Bound**
CT + MRI	7	3	7.785714286	8.753148189	1.855357143	15.00714286
CT + PET	17	4.2	22.74588235	30.0251711	9.279352941	37.99847059
EMG + MRI	2	9.5	9.5	3.132341619	5	14
EMG + PET	1	5	5	NA	NA	NA
MRI + PET	70	6	8.56	8.182681833	6.831321429	10.53789286
MRI + Ultrasound	1	3	3	NA	NA	NA
**Statistic**	**Degrees of Freedom**	**P Value**	**Method**			
1.274468338	3	0.7352045648	Kruskal-Wallis rank sum test			
**Comparison**	**Z Value**	**P Unadjusted**	**Bonferroni Adjusted P Value**			

CT + MRI vs. CT + PET	-0.8860029076	0.3756159604	1			
CT + MRI vs. EMG + MRI	-0.9736812002	0.3302148546	1			
CT + PET vs. EMG + MRI	-0.5120599214	0.6086090731	1			
CT + MRI vs. MRI + PET	-0.9082410545	0.3637508779	1			
CT + PET vs. MRI + PET	0.1400059322	0.8886553033	1			
EMG + MRI vs. MRI + PET	0.5865595432	0.5574995606	1			

Table S15: Kruskal-Wallis analysis of 3 diagnostic modalities in patients without biopsy (mutually exclusive) with respect to (A) Time From Symptom Onset to Diagnosis, (B) Time From Treatment 1 to Progression, (C) Survival Time from Diagnosis, (D) Survival Time from Symptom Onset.

15A

Symptom Onset to Diagnosis						
**Diagnostic Method**	**N**	**Median**	**Mean**	**Standard Deviation**	**Lower Bound**	**Upper Bound**
EMG + MRI + PET	1	24	24	NA	NA	NA

15B

Treatment 1 to Progression						
**Diagnostic Method**	**N**	**Median**	**Mean**	**Standard Deviation**	**Lower Bound**	**Upper Bound**
EMG + MRI + PET	1	6	6	NA	NA	NA

15C

Survival Time from Diagnosis						
**Diagnostic Method**	**N**	**Median**	**Mean**	**Standard Deviation**	**Lower Bound**	**Upper Bound**
CT + MRI + PET	2	5	5	2.814850675	1	9
EMG + MRI + PET	7	6	7.928571429	4.36284044	4.710714286	11.21607143
**Statistic**	**P Value**	**Method**				
4.5	0.5548679537	Wilcoxon rank sum test with continuity correction				

15D

Survival Time from Symptom Onset						
Diagnostic Method	N	Median	Mean	Standard Deviation	Lower Bound	Upper Bound
CT + MRI + PET	2	5	5	2.840872204	1	9
EMG + MRI + PET	7	9	11.35714286	8.306872874	5.714285714	18.07321429
Statistic	P Value	Method				
3.5	0.3777807422	Wilcoxon rank sum test with continuity correction				

Table S16. List of all publications included in this natural history analysis from which data were extracted.

Authors	Title	Journal	Year of Publication	Volume	Issue	Pages	DOI
Bourque PR, Sampaio ML, Warman-Chardon J, Samaan S, Torres C	Neurolymphomatosis of the lumbosacral plexus and its branches: case series and literature review	BMC Cancer	2019	19	1	1149	10.1186/s12885-019-6365-y
Bourque PR, Warman Chardon J, Bryanton M, Toupin M, Burns BF, Torres C	Neurolymphomatosis of the Brachial Plexus and its Branches: Case Series and Literature Review	Canadian Journal of Neurological Sciences	2018	45	2	137-143	10.1017/cjn.2017.282
Yaseen MT, Aziz PAA, Siddique K, Tariq TA	Neurolymphomatosis of the Sciatic and Tibial Nerves	Journal of College of Physicians and Surgeons Pakistan	2019	29	12	S86-S88	10.29271/jcpsp.2019.12.S86
DeBoer SR, Lesche S, Rodriguez FJ, Ostrow LW	Teaching NeuroImages: Neurolymphomatosis	Neurology	2019	93	12	e1229-e1230	10.1212/WNL.0000000000008131
DeVries AH, Howe BM, Spinner RJ, Broski SM	B-cell peripheral neurolymphomatosis: MRI and 18F-FDG PET/CT imaging characteristics	Skeletal Radiology	2019	48	7	1043-1050	10.1007/s00256-019-3145-3
Sato H, Hiroshima S, Anei R, Kamada K	Primary neurolymphomatosis of the trigeminal nerve	British Journal of Neurosurgery	2019	11		1-4	10.1080/02688697.2019.1568391
Grisariu S, Avni B, Batchelor TT, van den Bent MJ, Bokstein F, Schiff D, Kuittinen O et al.	Neurolymphomatosis: an International Primary CNS Lymphoma Collaborative Group report	Blood	2010	115	24	5005-5011	10.1182/blood-2009-12-258210
Brand C, Pala A, Scheuerle A, Scheglmann K, König R, Kratzer W et al.	Neurolymphomatose : Zwei Fallberichte [Neurolymphomatosis : Two case reports]	Der Nervenarzt	2018	89	6	701-704	10.1007/s00115-017-0460-6
Soni N, Ora M	FDG PET/CT in Neurolymphomatosis	Radiology	2021	298	1	36	10.1148/radiol.2020202835
Phang I, Craig-Mcquaide A, Kinch K, Taylor W	Burkitt's neurolymphomatosis of the trigeminal nerve	BMJ Case Reports	2018				10.1136/bcr-2018-225958
Kaulen LD, Foss FM, Fulbright RK, Huttner A, Baehring JM	Neurolymphomatosis of the thoracic sympathetic chain	Neurology	2017	89	18	1926-1927	10.1212/WNL.0000000000004600
Shree R, Goyal MK, Modi M, Gaspar BL, Radotra BD, Ahuja CK et al.	The Diagnostic Dilemma of Neurolymphomatosis	Journal of Clinical Neurology	2016	12	3	274-281	10.3988/jcn.2016.12.3.274

Khandelwal S, Saxena S, Hansalia DJ	Neurolymphomatosis: A Surreal Presentation of Lymphoma	Indian Journal of Medical and Paediatric Oncology	2017	38	2	289-290	10.4103/ijmpo.ijmpo_151_16
Baehring J, Cooper D	Neurolymphomatosis	Journal of Neuro-Oncology	2004	68	3	243-244	10.1023/b:neon.0000033496.29296.a7
Denays R, Baudrez V, Abouhamad P, Derème T, Milbouw G, Hermanne JP	Plexiform neurolymphomatosis	Revue Neurologique (Paris)	2016	172	4-5	328-330	10.1016/j.neurol.2016.02.008
Keddie S, Nagendran A, Cox T, Bomsztyk J, Jaunmuktane Z, Brandner S et al.	Peripheral nerve neurolymphomatosis: Clinical features, treatment, and outcomes	Muscle & Nerve	2020	62	5	617-625	10.1002/mus.27045
Iacobellis F, Di Serafino M, Blasio R, Barbuto L, Pezzullo F, Romano L	Secondary Neurolymphomatosis of the Radial Nerve: A Diagnostic Challenge	American Journal of Case Reports	2019	20		1652-1658	10.12659/AJCR.916961
Liu KC, Hennessey MA, McCall CM, Proia AD	Ocular involvement in neurolymphomatosis	American Journal of Ophthalmology Case Reports	2018	10		148-151	10.1016/j.ajoc.2018.02.023
Gan HK, Azad A, Cher L, Mitchell PL	Neurolymphomatosis: diagnosis, management, and outcomes in patients treated with rituximab	Neuro-Oncology	2010	12	2	212-215	10.1093/neuonc/nop021
Jeong J, Kim SW, Sung DH	Neurolymphomatosis: a single-center experience of neuromuscular manifestations, treatments, and outcomes	Journal of Neurology	2021	268	3	851-859	10.1007/s00415-020-10202-0
Diaz-Arrastia R, Younger DS, Hair L, Inghirami G, Hays AP, Knowles DM et al.	Neurolymphomatosis: a clinicopathologic syndrome re-emerges	Neurology	1992	42	6	1136-1141	10.1212/wnl.42.6.1136
van den Bent MJ, de Bruin HG, Beun GD, Vecht CJ	Neurolymphomatosis of the median nerve	Neurology	1995	45	7	1403-1405	10.1212/wnl.45.7.1403
Chang GY	Evolution of Neurolymphomatosis to Lymphomatosis Cerebri	Journal of Clinical Neurology	2017	13	2	203-204	10.3988/jcn.2017.13.2.203
Sasaki R, Ohta Y, Yamada Y, Tadokoro K, Takahashi Y, Sato K et al.	Neurolymphomatosis in the Cauda Equina Diagnosed by an Open Biopsy	Internal Medicine	2018	57	23	3463-3465	10.2169/internalmedicine.1049-18
Jiménez Zapata HD, Rojas Medina LM, Carrasco Moro R, Martínez Rodrigo A, García-Cosio Piqueras M	Cauda equina syndrome secondary to neurolymphomatosis: Case report and literature review	Neurocirugia	2018	29	3	138-142	10.1016/j.neucir.2017.07.001
Li H, Luo SS, Zhao CB	Neurolymphomatosis caused by diffuse large B-cell lymphoma presenting as isolated brachial plexopathy	Chinese Medical Journal	2019	132	22	2762-2764	10.1097/CM9.0000000000000514
Pan Q, Luo Y	Recurrence of nasal type NK/T cell lymphoma presenting as neurolymphomatosis on 18F-FDG PET/CT: A case report and literature review	Medicine (Baltimore)	2020	99	1	e18640	10.1097/MD.0000000000018640
Kobayashi H, Abe Y, Miura D, Narita K, Kitadate A, Takeuchi M et al.	Limited efficacy of high-dose methotrexate in patients with neurolymphomatosis	International Journal of Hematology	2019	109	3	286-291	10.1007/s12185-018-02586-7
Sosa-Albacete F, Pappolla A, Hem S, Kohan D, Otero V, Zurru-Ganen MC et al.	Neurolinfomatosis primaria de cola de caballo como manifestacion inicial del virus de la	Revue Neurologique	2019	69	7	301-302	10.33588/rn.6907.2019244

	inmunodeficiencia humana [Primary neurolymphomatosis in the cauda equina as the initial symptom of human immunodeficiency virus]						
Murthy NK, Hébert-Blouin MN, Capek S, Prasad NK, Amrami KK, Spinner RJ	Understanding the Pathognomonic Tumefactive Appearance of Neurolymphomatosis: A Unifying Theory of Neurolymphoma	World Neurosurgery	2020	141		e490-e497	10.1016/j.wneu.2020.05.228
Sideras PA, Matthews J, Sakib SM, Ofikwu F, Spektor V	Neurolymphomatosis of the peripheral nervous system: a case report and review of the literature	Clinical Imaging	2016	40	6	1253-1256	10.1016/j.clinimag.2016.08.014
Narita K, Kobayashi H, Kitadate A, Abe Y, Miura D, Takeuchi M et al.	Neurolymphomatosis of the sciatic and tibial nerves as an initial presentation of lung diffuse large B cell lymphoma detected by positron emission tomography/computed tomography	International Journal of Hematology	2019	110	4	385-386	10.1007/s12185-019-02705-y
Tanimizu M, Kobayashi K	Neurolymphomatosis Appeared Following Primary Central Nervous System Lymphoma	Internal Medicine	2017	56	4	463-464	10.2169/internalmedicine.56.7238
Padilha IG, Fischer T, Pacheco FT, Da Rocha AJ	Brachial and sacral plexus neurolymphomatosis - unusual regions for disease relapses	Arquivos de Neuro-Psiquiatria	2019	77	11	832-833	10.1590/0004-282X20190104
Negre Busó M, Balliu Collgrós E, Rubió Rodríguez A, Peláez Hernández I, Mestre Fusco A, Roncero Vidal JM	Utility of the 18F-FDG PET/CT in the diagnosis of the neurolymphomatosis: A case report	Revista Española de Medicina Nuclear e Imagen Molecular	2021	40	5	328-331	10.1016/j.remnie.2020.09.017
Sheng S, Sharma R, Samant R, Yuan J, Nalleballe K, Kapoor N	Isolated primary neurolymphomatosis with cranial multineuritis: a case presentation	Neurological Sciences	2021	42	3	1223-1226	10.1007/s10072-020-04806-7
Li V, Jaunmuktane Z, Cwynarski K, Carr A	Diagnostic delay in a case of T-cell neurolymphomatosis	BMJ Case Reports	2019	12	12	e232538	10.1136/bcr-2019-232538
Liu Z, Jiang T, Hou N, Jia Y	Peripheral neurolymphomatosis with tracheal asphyxia: a case report and literature review	BMC Neurology	2015	15		149	10.1186/s12883-015-0405-2
Barahona D, Adlerstein I, Donoso J, Mercado F	Sciatic and median nerve neurolymphomatosis as initial presentation of B-cell Lymphoma	Radiologia	2020	S0033-8338	20	30171-30175	10.1016/j.rx.2020.10.008
Dakwar E, Teja S, Alleyne CH Jr	Sciatic neurolymphomatosis	Neurology	2004	63	9	1751	10.1212/01.wnl.0000138501.06028.68
Campagnolo M, Cacciavillani M, Cavallaro T, Ferrari S, Gasparotti R, Zambello R et al.	Neurolymphomatosis, a rare manifestation of peripheral nerve involvement in lymphomas: Suggestive features and diagnostic challenges	Journal of the Peripheral Nervous System	2020	25	3	312-315	10.1111/jns.12401
Ganeshalingam R, Roach P, Schembri GP	Diffuse Large B-Cell Lymphoma Recurring as Neurolymphomatosis on FDG PET/CT	Clinical Nuclear Medicine	2019	44	2	145-147	10.1097/RLU.0000000000002388

Gorospe L, Gallego-Rivera JI, Rioja-Martín ME, Chinea-Rodríguez A	Bilateral sciatic nerve neurolymphomatosis: PET/CT findings	Annals of Hematology	2017	96	6	1059-1060	10.1007/s00277-017-2988-8
Puffer RC, Dyck PJB, Paul P, Broski SM, Amrami KK, Spinner RJ	Putative mechanisms for spread and transformation of cutaneous T-cell lymphoma to neurolymphomatosis	Muscle & Nerve	2019	60	5	E30-E33	10.1002/mus.26670
Shima T, Tsujino A	[Neurolymphomatosis Mimicking Inflammatory Neuropathy with Slight FDG Uptake on PET/CT and Distinct MRI Abnormalities in the Brachial Plexus: A Case Report]	Brain and Nerve	2020	72	9	987-992	10.11477/mf.1416201632
de Vaugelade C, de Clermont-Gallerande H, Meyer M, My Duyen Tiphaine A, Benjamin A, Ducassou S, Chateil JF et al.	FDG PET/CT in Pediatric Neurolymphomatosis	Clinical Nuclear Medicine	2017	42	5	e255-e257	10.1097/RLU.0000000000001624
Haydaroglu Sahin H, Mete A, Pehlivan M	Neurolymphomatosis in non-Hodgkin lymphoma with cranial multineuritis: A case report	Medicine (Baltimore)	2018	97	15	e0303	10.1097/MD.0000000000010303
Uematsu N, Sumi M, Kaiume H, Takeda W, Kirihara T, Ueki T et al.	[Neurolymphomatosis due to enteropathy-associated T-cell lymphoma clinically diagnosed by FDG-PET/CT and subsequently confirmed by autopsy]	Rinsho Ketsueki	2018	59	1	69-74	10.11406/rinketsu.59.69
Tai R, Maingard J, Nambiar M, Lim K	High-grade B-cell lymphoma relapse presenting as neurolymphomatosis of the median nerve	BMJ Case Reports	2019	12	3	e228742	10.1136/bcr-2018-228742
Shimizu I, Hamano Y, Sato S, Takeda W, Kirihara T, Sato K et al.	Neurolymphomatosis in a patient with extranodal NK/T-cell lymphoma, nasal-type: a case report and literature review	Internal Medicine	2014	53	5	471-475	10.2169/internalmedicine.53.0262
Tomita M, Koike H, Kawagashira Y, Iijima M, Adachi H, Taguchi J et al.	Clinicopathological features of neuropathy associated with lymphoma	Brain	2013	136	Pt. 8	2563-2578	10.1093/brain/awt193
Fatima N, Zaman MU, Zaman A, Zaman SU	Neurolymphomatosis - Rare presentation in non-Hodgkin's lymphoma: The role of 18F-fluorodeoxyglucose positron-emission tomography and computerized tomography imaging	World Journal of Nuclear Medicine	2020	19	2	159-161	10.4103/wjnm.WJNM_22_19
Murthy NK, Amrami KK, Spinner RJ	Circumdural extension of perineural spread leading to bilateral disease in neurolymphomatosis	Acta Neurochir (Wien)	2020	162	12	3197-3200	10.1007/s00701-020-04417-3
Guberman A, Rosenbaum H, Braciale T, Schlaepfer WW	Human neurolymphomatosis	Journal of the Neurological Sciences	1978	36	1	1-12	10.1016/0022-510x(78)90157-0
Yi FF, Luo SS, Zhu WH, Zhao CB	Neurolymphomatosis Caused by Nasal-type Extranodal Natural Killer/T-cell Lymphoma	Chinese Medical Journal	2017	130	5	625-626	10.4103/0366-6999.200547
Facchinelli D, Ciliberti E, Stüssi G, Ceriani L, Zucca E	Sciatic pain by neurolymphomatosis as initial presentation of disseminated diffuse large B cell lymphoma involving the testis and the CNS	Hematological Oncology	2020	38	2	197-200	10.1002/hon.2698

Lorance DK, Allison JB, Gardner JA, Waheed W	Protean Manifestations and Diagnostic Challenges Including Discordance Between Electrodiagnostic-Radiologic Studies in Neurolymphomatosis	The Neurologist	2020	25	1	4-9	10.1097/NRL.0000000000000245
Nepal P, Batchala PP, Rehm PK, Fadul CE	Diffuse large B-cell lymphoma relapse presenting as extensive neurolymphomatosis	Journal of Neuroradiology	2020	33	3	230-235	10.1177/1971400920924799
Byun JM, Kim KH, Kim M, Kim TM, Jeon YK, Park JH et al.	Diagnosis of secondary peripheral neurolymphomatosis: a multi-center experience	Leukemia & Lymphoma	2017	58	11	2624-2632	10.1080/10428194.2017.1312376
Duchesne M, Roussellet O, Maisonobe T, Gachard N, Rizzo D, Armand M et al.	Pathology of Nerve Biopsy and Diagnostic Yield of PCR-Based Clonality Testing in Neurolymphomatosis	Journal of Neuropathology & Experimental Neurology	2018	77	9	769-781	10.1093/jnen/nly055
Khader A, Vineetha M, George M, Manakkad SP, Balakrishnan S, Rajan U	Neurolymphomatosis in Primary Cutaneous CD4+ Pleomorphic Small/Medium-sized T-cell Lymphoma Mimicking Hansen's Disease	Indian Journal of Dermatology	2017	62	3	315-317	10.4103/ijd.IJD_553_16
Park HJ, Shin HY, Kim SH, Jeong HN, Choi YC, Suh BC et al.	Partial Conduction Block as an Early Nerve Conduction Finding in Neurolymphomatosis	Journal of Clinical Neurology	2018	14	1	73-80	10.3988/jcn.2018.14.1.73
Kobayashi M, Sakai Y, Kariya Y, Sakai H, Hineno A, Oyanagi K et al.	First pathological report of a de novo CD5-positive diffuse large B-cell lymphoma patient presenting with Guillain-Barré syndrome-like neuropathy due to neurolymphomatosis	Neuropathology	2018				10.1111/neup.12470
Yu Y, Ren M, Qi X	Pathologically proven peripheral neurolymphomatosis	Neurology India	2016	64	4	805-807	10.4103/0028-3886.185354
Nishikawara M, Kawakami T, Sakai H, Kawakami F, Nishina S, Uehara T et al.	Magnetic Resonance Imaging-negative, Rituximab-resistant Neurolymphomatosis as a Paradoxical Presentation of Relapsed Primary Adrenal Lymphoma	Internal Medicine	2020	59	11	1437-1443	10.2169/internalmedicine.4085-19
Fitzgerald L, Stephens DM	Burkitt Lymphoma Presenting as Cranial Multineuritis Secondary to Primary Neurolymphomatosis: A Diagnostic Challenge	Clinical Lymphoma, Myeloma and Leukemia	2020	20	4	e201-e204	10.1016/j.clml.2020.01.012
Mahmoud RA, Abrams CK	Acute demyelinating neuropathy in a patient with neurolymphomatosis	BMJ Case Reports	2018				10.1136/bcr-2017-222814
Asanome A, Kano K, Takahashi K, Saito T, Sawada J, Katayama T	[A case of neurolymphomatosis that was diagnosed by acoustic nerve biopsy]	Rinsho Shinkeigaku	2018	58	2	93-99	10.5692/clinicalneurol.cn-001080
Rai W, Olcese V, Elsheikh B, Stino AM	Horner's Syndrome as Initial Manifestation of Possible Brachial Plexopathy Neurolymphomatosis.	Frontiers in Neurology	2019	10		4	10.3389/fneur.2019.00004
Chague P, Phan CM, Lapusan S, Zhang-Yin J, Cottereau AS	18F-FDG PET/CT imaging findings of extensive neurolymphomatosis as a relapse of diffuse large B cell lymphoma	Diagnostic and Interventional Imaging	2019	100	9	527-528	10.1016/j.diii.2019.02.006
Odabasi Z, Parrott JH, Reddy VV, Oh SJ	Neurolymphomatosis associated with muscle and cerebral involvement caused by natural	Journal of the Peripheral Nervous System	2001	6	4	197-203	10.1046/j.1529-

	killer cell lymphoma: a case report and review of literature						8027.2001.01018.x
Usami M, Murase K, Takada K, Iijima K, Yoshida M, Tatekoshi A et al.	[Neurolymphomatosis of the sciatic nerve diagnosed by FDG-PET/CT]	Rinsho Ketsueki	2016	57	1	52-55	10.11406/rinketsu.57.52
Konishi H, Taguchi Y, Yamamoto M, Nukui T, Dougu N, Nakatsuji Y	[A case of neurolymphomatosis presented as cauda equine syndrome accompanied with M-proteinemia]	Rinsho Shinkei-gaku	2018	58	4	223-228	10.5692/clinicalneurol.cn-001079
Singh SS, Mittal BR, Kumar R, Singh H, Balaini N, Goyal M	Primary Central Nervous System Lymphoma With Diffuse Neurolymphomatosis Involving Multiple Cranial and Spinal Nerve Roots	Clinical Nuclear Medicine	2020	45	6	e285-e287	10.1097/RLU.0000000000003018
Davidson T, Kedmi M, Avigdor A, Komisar O, Chikman B, Lidar M et al.	FDG PET-CT evaluation in neurolymphomatosis: imaging characteristics and clinical outcomes	Leukemia & Lymphoma	2018	59	2	348-356	10.1080/10428194.2017.1352096
Daher A, Kamiya-Matsuoka C, Woodman K	Patient With 2 Hematologic Malignancies Presenting as Neurolymphomatosis	Journal of Clinical Neuromuscular Disease	2018	19	3	124-130	10.1097/CND.0000000000000183
Matsue K, Abe Y, Narita K, Kobayashi H, Kitadate A, Takeuchi M et al.	Diagnosis of intravascular large B cell lymphoma: novel insights into clinicopathological features from 42 patients at a single institution over 20 years	British Journal of Haematology	2019	187	3	328-336	10.1111/bjh.16081
Fritzhand SJ, Esmaeli B, Sun J, Debnam JM	Primary disease sites and patterns of spread in cases of neurolymphomatosis in the orbit associated with lymphoma	Cancer Imaging	2021	21	1	39	10.1186/s40644-021-00409-3
Switlyk MD, Skeie AT, Lund-Iversen M, Østenstad B	Magnetic resonance imaging and 18F-fluorodeoxyglucose positron emission tomography/computed tomography findings in neurolymphomatosis: an uncommon presentation of diffuse large B cell lymphoma	Annals of Hematology	2020	99	1	203-205	10.1007/s00277-019-03850-4
Biswal CK, Mittal BR, Shukla J, Vatsa R, Bhattacharya A, Prabhakar S	Fluorothymidine PET/CT in neurolymphomatosis	Clinical Nuclear Medicine	2015	40	5	e290-e292	10.1097/RLU.0000000000000632
Lim AT, Clucas D, Khoo C, Parameswaran BK, Lau E	Neurolymphomatosis: MRI and (18) FDG-PET features	Journal of Medical Imaging and Radiation Oncology	2016	60	1	92-95	10.1111/1754-9485.12321
Gykiere P, Jans L, Degrieck B, Goethals I	Neurolymphomatosis on 18F-FDG PET/CT: Diagnosis and Therapy Response	Clinical Nuclear Medicine	2016	41	2	142-143	10.1097/RLU.0000000000000982
Briani C, Visentin A, Cavallaro T, Cacciavillani M, Cabrini I, Ferrari S et al.	Primary neurolymphomatosis as clinical onset of chronic lymphocytic leukemia	Annals of Hematology	2017	96	1	159-161	10.1007/s00277-016-2852-2
Kamiya-Matsuoka C, Shroff S, Gildersleeve K, Hormozdi B, Manning JT, Woodman KH	Neurolymphomatosis: a case series of clinical manifestations, treatments, and outcomes	Journal of the Neurological Sciences	2014	343	1-2	144-148	10.1016/j.jns.2014.05.058
Lagarde S, Tabouret E, Matta M, Franques J, Attarian S, Pouget J et al.	Primary neurolymphomatosis diagnosis and treatment: a retrospective study	Journal of the Neurological Sciences	2014	342	1-2	178-181	10.1016/j.jns.2014.04.030

Santos E, Scolding NJ	Neurolymphomatosis mimicking neurosarcoidosis: a case report	Journal of Medical Case Reports	2010	4		5	10.1186/1752-1947-4-5
Aboueldahab N, Shafik MA, Megahed A, Vesselle H	Neurolymphomatosis; a case report	Clinical Imaging	2021	80		329-333	10.1016/j.clinimag.2021.08.016
Choi YJ, Shin JA, Kim YH, Cha SJ, Cho JY, Kang SH et al.	Neurolymphomatosis of Brachial Plexus in Patients with Non-Hodgkin's Lymphoma	Case Reports in Oncological Medicine	2013		492329		10.1155/2013/492329
Ogawa S, Fukunaga A, Kanata M, Kikuzawa A, Bito T, Otsuka Y et al.	Neurolymphomatosis associated with erythrodermic mycosis fungoides	Acta Dermato-Venereologica	2014	94	2	227-228	10.2340/00015555-1684
Hong RS, Woodson EA, Hansen MR	Neurolymphomatosis mimicking chemotherapy-induced ototoxicity	Otology & Neurotology	2009	30	4	566-569	10.1097/MAO.0b013e3181a527b2
Bund C, Heimburger C, Trensz P, Fohrer C, Kremer S, Namer IJ	FDG PET to Diagnose Neurolymphomatosis in a Case of Triple-Hit B-Cell Lymphoma	Clinical Nuclear Medicine	2017	42	6	458-460	10.1097/RLU.0000000000001608
Pham M, Awad M	Lymphoma relapse presenting as neurolymphomatosis	Asian Journal of Neurosurgery	2016	11	1	73	10.4103/1793-5482.165783
Salm LP, Van der Hiel B, Stokkel MP	Neurolymphomatosis diagnosed by (18)F-FDG PET-CT	Clinical Nuclear Medicine	2013	38	6	e261-e262	10.1097/RLU.0b013e318266ce70
Seegobin K, Alhaj Moustafa M, Fischer D, Keller K, Hastings J, Kharfan-Dabaja MA et al.	Systemic ALK-positive anaplastic large cell lymphoma with bilateral optic neurolymphomatosis resulting in permanent blindness	Clinical Case Reports	2020	8	12	2629-2633	10.1002/ccr3.3231
Le Guennec L, Maisonobe T, Choquet S, Massein A, Azoulay-Cayla A, Villain N et al.	Neurolymphomatosis as a relapse of primary cerebral nervous system lymphoma	Leukemia & Lymphoma	2017	58	3	729-731	10.1080/10428194.2016.1211277
Wan MY, Ardeshna KM, Bomanji J	Neurolymphomatosis in a patient with lymphoblastic lymphoma	British Journal of Haematology	2012	156	6	691	10.1111/j.1365-2141.2011.09002.x
Bjornard KL, Leventaki V, Nichols KE, Sandlund JT, Prockop S, Ehrhardt MJ	Two-year-old female with EBV-positive diffuse large B-cell lymphoma and subsequent CNS involvement with neurolymphomatosis	Pediatric Blood & Cancer	2018	65	12	e27415	10.1002/pbc.27415
Ramírez Ocaña D, Gutiérrez Cardo AL, González Díaz L, Hurst K, Espeso de Haro M	Neurolinfomatosis como manifestación inicial de recidiva en linfoma [Neurolymphomatosis as initial manifestation of recurrence in lymphoma]	Revista Española de Medicina Nuclear e Imagen Molecular	2014	33	1	50-51	10.1016/j.remn.2013.02.003
Abad S, Zagdanski AM, Brechignac S, Thioliere B, Brouet JC, Mariette X	Neurolymphomatosis in Waldenström's macroglobulinaemia	British Journal of Haematology	1999	106	1	100-103	10.1046/j.1365-2141.1999.01482.x
Brandstadter R, Brody J, Morgello S, Motiwala R, Shin S, Lublin F et al.	Primary Neurolymphomatosis Presenting With Polyradiculoneuropathy Affecting One Lower Limb	Journal of Clinical Neuromuscular Disease	2015	17	1	6-12	10.1097/CND.0000000000000088
Campagnolo M, Cacciavillani M, Briani C	Heterogeneous clinical and imaging findings and long-term prognosis in patients with neurolymphomatosis	Muscle & Nerve	2021	63	3	e24-e26	10.1002/mus.27154

Awis Qarni F, Tai E, Wh WH, Husin A	Homonymous Hemianopia: A Rare Presentation of Secondary Central Nervous System Neurolymphomatosis	The Cureus Journal of Medical Science	2018	10	5	e2708	10.7759/cureus.2708
Trelles JO, Trelles L	La neurolymphomatose périphérique humaine [Human peripheral neurolymphomatosis]	Revue Neurologique (Paris)	1983	139	12	703-714	
Hong CM, Lee SW, Lee HJ, Song BI, Kim HW, Kang S	Neurolymphomatosis on F-18 FDG PET/CT and MRI Findings: A Case Report	Nuclear Medicine and Molecular Imaging	2011	45	1	76-78	10.1007/s13139-010-0070-8
Nishida K, Takenaka Y, Yamasaki H, Futamura N	Garcin Syndrome in a Patient With Neurolymphomatosis	Neurology	2021				10.1212/WNL.0000000000012485
Bower SP, McKelvie P, Peppard RW, Roberts L	Neurolymphomatosis presenting as mononeuritis multiplex	Journal of Clinical Neuroscience	1999	6	6	530-532	10.1016/s0967-5868(99)90020-6
Trevisan AC, Ribeiro FB, Itikawa EN, Alexandre LS, Pitella FA, Santos AC et al.	18F-FDG PET/CT/MRI Fusion Images Showing Cranial and Peripheral Nerve Involvement in Neurolymphomatosis	Indian Journal of Nuclear Medicine	2017	32	1	77-78	10.4103/0972-3919.198502
Bezier M, Reguiaï Z, Delaby P, Laroche L, Saïd G, Bernard P et al.	Neurolymphomatosis associated with Sézary syndrome	Archives of Dermatological Research	2009	145	3	294-296	10.1001/archdermatol.2008.584
Alazawi S, Elomri H, Taha R, Bakr M, Abdelhamid MT, Szabados L et al.	Neurolymphomatosis of the median nerve, optic nerve, L4 spinal nerve root and cauda equina in patients with B-cell malignancies: a case series	Journal of Medical Case Reports	2021	15	1	133	10.1186/s13256-021-02714-8
Started at article 126							
Akagi A, Ono K, Hamaguchi T, Samuraki M, Nakada M, Shima Y, Oohata T, Yamada M et al.	Neurolymphomatosis exhibiting repeated exacerbation and remission in both the peripheral and central nervous system	Journal of the Neurological Sciences	2014	345	1-2	267-268	10.1016/j.jns.2014.07.038
Borit A, Altrocchi P	Recurrent Polyneuropathy and Neurolymphomatosis	JAMA Network - Arch Neuro	1971	24			
Bruce D, Eagleton H, Subesinghe M	Diagnostic and response assessment FDG PET-CT in neurolymphomatosis	Clinical Case Reports	2016	12	4	1172-1174	10.1002/ccr3.734
Cassellberry J, Kritz A	Neurolymphomatosis: A Case Study of Diffuse Large B-Cell Lymphoma	Clinical Journal of Oncology Nursing	2012	16	6	636-637	10.1188/12.CJON.636-637
Cheung C, Lopes D, Hung KN, Chan T, Chan KW, Kwong YL	Neurolymphomatosis: role of positron emission tomography in diagnosis		2011			1313-1314	10.1007/s00277-011-1379-9
Jong A, Mous R, Dongen GAMS, Hoekstra OS, Nievelstein RAJ, Keizer B	Zr-rituximab PET/CT to detect neurolymphomatosis	American Journal of Hematology	2016	91	6	649-650	10.1002/ajh.24328
Durán C, Infante JR, Serrano J, Rayo JI, Garcı ́a I, Sanchez MLD	Neurolymphomatosis: Diagnosis of extension and assessment of response to treatment with PET-CT	Revista Espanola de Medicina Nuclear	2009	28	6	295-298	10.1016/j.remn.2009.07.002
Hanna R, Primio GAD, Schweitzer M, Torres C, Sheikh Adnan, Chakraborty S	Progressive neurolymphoma with cutaneous disease: Response in a patient with mycosis fungoides	Springer	2013	42	1	1011-1015	10.1007/s00256-013-1595-6

Kajáry K, Molnár Z, Mikó I, Barsi P, Lengyel Z, Szakáll Jr S	Neurolymphomatosis as a late relapse of non-Hodgkin's lymphoma detected by F-18 FDG PET/CT: A case report		2013	33	1	39-42	
Kuroda Y, Naata H, Kakigi R, Oda K, Shibasaki H, Nakashiro H	Human neurolymphomatosis by adult T-cell leukemia	Neurology	1989	39	1	144-146	
Lahoria R, Dyck P, Macon W, Crum B, Spinner R, Amrami K, Zeldenrust S, Tracy J	Neurolymphomatosis: A Report of 2 Cases Representing Opposite Ends of the Clinical Spectrum	Muscle & Nerve	2015	52	1	449-454	10.1002/mus.24646
Miki M, Masaki Y, Nakamura T, Iwao H, Nakajima A, Sakai T, Sawaki T, Kawanami T, Karro M, Kurose N, Fujita Y, Tanaka M, Fukushima T, Hirose Y, Umehara H	Primary neurolymphomatosis of the cervical nerve root	The Japanese Journal of Clinical Hematology	2010	51	7	564-567	
Nishizawa M, Yamashita K, Nakamoto Y, Kotani S, Kondo T, Takaori-Kondo A	Neurolymphomatosis as a manifestation of relapsed primary cardiac lymphoma	Images in Hematology	2010	92	1	679-680	10.1007/s12185-010-0716-4
Okada M, Takamatsu K, Naoto O, Nakamura H	Solitary neurolymphomatosis of the brachial plexus mimicking benign nerve sheath tumour: case report	British Journal of Neurosurgery	2013	27	3	386-387	10.3109/02688697.2012.737959
Sakai N, Ito-Yamashita T, Takahashi G, Baba S, Koizumi S, Yamasaki T, Tokuyama T, Namba H	Primary Neruolymphomatosis of the Lower Cranial Nerves Presenting as Dysphagia and Hoarseness: A Case Report	Neurolymphomatosis of the Lower Cranial Nerves	2014	75		e62-e66	10.1055/2-0033-1363505
Sugai A, Konno T, Yano T, Umeda M, Oyake M, Fujita N	Neurolymphomatosis presenting as bilateral tongue atrophy: A case report	Clinical Neurology Journal	2012	52	8	589-591	
Umeda M, Kondo T, Nishikori M, Kitano T, Hishizawa M, Kadowaki N, Takaori-Kondo A	A case of neurolymphomatosis caused by follicular lymphoma successfully treated with bendamustine	Clinical Case Reports	2016	4	1	23-25	10.1002/ccr3.436
Vecchio D, Mittino D, Terazzi E, Luca N, Conconi A, Monaco F	A case of cranial multinevritis: from the onset to the diagnosis of primary neurolymphomatosis	BMJ Case Reports	2012			1 - 3	10.1136/bcr.06.2011.4299
Wang Y, Wang JC, Jiang C, Peng Q, Wu M, Ma X	Secondary neurolymphomatosis of spinal nerve roots detected by F-FDG PET/CT: a case report and differential diagnosis of the case	Hellenic Journal of Nuclear Medicine	2015	18	3	261-263	
Zhou WL, Wu HB, Weng CS, Han YJ, Wang M, Huang S, Wang QS	Usefulness of F-FDG PET/CT in the detection of neurolymphomatosis	Nuclear Medicine Communications	2014	35	11	1107-1111	10.1097/MNM.0000000000000181
Tachibana T, Tomita N, Ueda T, Katoh J, Takemura S, Taguchi J, Suzuki Y, Kasahara M, Ishigatsubo Y, Fujita H	Systemic neurolymhomatosis complicated in diffuse large B-cell lymphoma	The Japanese Journal of Clinical Hematology	2007	48	12	1563-1566	
Canh NX, Tan NV, Tung TT, Son NT, Maurea S	F-FDG PET/CT in Neurolymphomatosis: Report of 3 Cases	Asia Oceania Journal of Nuclear Medicine & Biology	2014	2	1	57-64	
Chamberlain M, Fink J	Neurolymphomatosis: a rare metastatic complication of iffuse large B-Cell lymphoma	Journal of Neuro-oncology	2009	95		285-288	10.1007/s11060-009-9918-0

Czepczyński R, Guzikowska-Ruszkowska I, Sowiński J	Neurolymphomatosis detected by F-FDG PET/CT scan - a case report	Nuclear Medicine Review	2008	11	2	73-75	
Foo TL, Tak R, Puhaindran M	Peripheral Nerve Lymphomatosis	The Journal of Hand Surgery (Asian-Pacific Volume)	2017	22	1	104-107	10.1142/S021 88104177200 42
Guberman A	Fatal Peripheral Neurolymphomatosis	Neurology	1984				
Gutiérrez-Martínez A, Malo-de-Molina R, García-García N	Neuropatía craneal múltiple como manifestación de una neurolinfomatosis primaria	Revista de Neurología	2015	61	8	381-383	
Khong P, Pitham T, Owler B	Isolated Neurolymphomatosis of the Cauda Equina and Filum Terminale	SPINE	2008	33	21	e807-e811	
Kinoshita H, Yamakado H, Kitano T, Kitamura A, Yamashita H, Miyamoto M, Hitomi T, Okada T, Nakamoto Y, Sawamoto N, Takaori-Kondo A, Takahashi R	Diagnostic utility of FDG-PET in neurolymphomatosis: report of five cases	Journal of Neurology	2016	263		1719-1726	10.1007/s004 15-016-8190-4
Koyama T, O'uchi T, Matsue K	Neurolymphomatosis involving the trigeminal nerve and deep peroneal nerve in a patient with relapsed intravascular large B-cell lymphoma	European Journal of Haematology		85		275-276	10.1111/j.160 0-0609.2010.01 477.x
Lin M, Kilanowska J, Taper J, Chu J	Neurolymphomatosis - diagnosis and assessment of treatment response by FDG PET-CT	Hematological Oncology	2008	26		43-45	10.1002/hon. 837
Makranz C, Arkadir D, Nachmias B, Gatt M, Eliahou R, Atlan K, Mordechai A, Goldshmit N, Lossos A	Neurological misdiagnoses of lymphoma	Neurological Sciences	2021	42		1933-1940	10.1007/s100 72-020-04724-8
Moore K, Blumenthal D, Smith A, Ward J	Neurolymphomatosis of the lumbar plexus: High-resolution MR neurography findings	Neurology	2001			740-742	
Nishio M, Tamaki T, Ochi H, Shibamoto Y	Intraspinal Canal Neurolymphomatosis Detected by FDG-PET/CT	Clinical Nuclear Medicine	2009	34	9	610-612	
Rosso S, de Bruin H, Wu KL, van den Bent M	Diagnosis of neurolymphomatosis with FDG PET	Neurology	2006			722-723	
Roy SG, Parida GK, Tripathy S, Das CJ, Kumar R	Active Leprosy Neuritis Detected on FDG PET/CT	Clinical Nuclear Medicine	2018	43	2	132-133	10.1097/RLU .00000000000 01946
Sato M, Furuta M, Hirayanagi K, Nagamine S, Makioka K, Ikeda Y	A case of neurolymphomatosis presenting extended involvement of spinal nerve roots	Clinical Neurology Journal	2015	55		333-338	
Schuster N, Volney S, Kamdar M	Sciatic Lymphoma Mimicking Lumbar Radiculopathy	Pain Medicine	2018	19		2091-2092	10.1093/pm/ pny153
Shibata-Hamaguchi A, Samuraki M, Furui E, Ishida C, Kitagawa S, Nakao S, Minato H, Yamada M	B-cell neurolymphomatosis confined to the peripheral nervous system	Journal of the Neurological Sciences	2007	260		249-252	

Shoenfeld Y, Aderka D, Sandbank U, Gadot N, Santo M, Pinkhas J	Fatal peripheral neurolymphomatosis after remission of histiocytic lymphoma	NEUROLOGY	1983	33		243-245	
Sunami Y, Gotoh A, Hamano Y, Yahata Y, Sakurai H, Shirane S, Edahiro Y, Komatsu N	Various Neurological Symptoms by Neurolymphomatosis as the Initial Presentation of Primary Testicular Lymphoma	Case Reports in Oncology	2015	8		200-204	10.1159/000381874
Tanaka H, Yoshino K, Sakaida E, Hashimoto S, Takeda Y, Kawajiri C, Takagi T, Nakaseko C	Secondary Neurolymphomatosis Detected by Whole-Body Diffusion-Weighted Magnetic Resonance Imaging: A Case Report	Journal of Clinical and Experimental Hematopathology	2013	53	3	221-226	
Tsai HH, Chen YF, Hsieh ST, Chao CC	Neurolymphomatosis as the primary presentation of non-Hodgkin's Lymphoma	Journal of Neurology, Neurosurgery, and Psychiatry	2014	0	0	1 - 2 .	10.1136/innp-2014-308244
Ebihara Y, Ishii N, Akizuki K, Taniguchi A, Mochizuki H, Inatsu A Shiomi K, Nagamachi S, Nakazato M	A case of neurolymphomatosis: peripheral neuropathy induced by diffuse large B-cell lymphoma without any abnormal accumulation observed on early positron emission tomography-computed tomography	Brain and Nerve	2015	67	2	219-223	10.11477/mf.1416200116
Advani P, Jiang L, Srinivasan S, Foran J	Disseminated extranodal marginal zone lymphoma involving the gastocnemius muscle with sural neurolymphomatosis	Annals of Hematology	2015	94		1939-1940	10.1007/s00277-015-2453-5
Bllioskas S, Tsaligopoulos M, Kyriafinis G, Psillas G, Markou K, Perifanis V, Kouskouras K, Vital V	Bilateral secondary neurolymphomatosis of the internal auditory canal nerves: A case report	American Journal of Otolaryngology	2013	34		556-558	10.1016/j.amjoto.2013.04.002
Chaturvedi A, Singh J, Rastogi V	MRI diagnosis of neurolymphomatosis of the brachial plexus	Neurology India	2008	56	4		
Grisold W, Jellinger K, Lutz D	Human neurolymphomatosis in a patient with chronic lymphatic leukemia	Clinical Neuropathology	1990	9	5	224-230	
Groth CL, Nevel KS, Gwathmey KG, Bafakih F, Jones DE	Splenic Marginal Zone Lymphoma: An Indolent Malignancy Leading to the Development of Neurolymphomatosis	Muscle & Nerve	2017	55		440-444	10.1002/mus25404
Boasquevisque GS, Guidoni J, Moreira de Souza LA, Goncalves PG, Andrade CV, Pedras FV, Boasquevisque EM, Boasquevisque ETS	Bilateral Vagus Nerve Neurolymphomatosis Diagnosed Using PET/CT and Diffusion-Weighted MRI	Clinical Nuclear Medicine	2012	37	9	e225-e228	
Hashizume Y	Pathology of intravascular lymphoma	Brain and Nerve	2011	63	5	459-466	
Hung MH, Gau JP	Relapsed testicular lymphoma presenting with cranial nerve neurolymphomatosis	Neuroloy	2011	76		1441	10.1212/WNL.0b013e318216711a
Jung YH, Woo IS, Han DJ, Han CW	F-fluoro-2deoxy-D-glucose positron emission tomogrpahy findings of neurolymphomatosis	Images of Hematology	2014	49	2	83	10.5045/br.2014.49.2.83
Koike H, Katsuno M, Sobue G	New teased-fibre definition represent specific mechanisms of neuropathy	Journal of Neurology, Neurosurgery, and Psychiatry	2018	0	1	1	10.1136/jnnp-2018-319493

Kosa SC, Peller PJ, Klein CJ	T-cell neurolymphomatosis involving cauda equina and sciatic nerves	Neurology	2009	72		98	10.1212/01.wnl.0000338598.07063.5b
Oner AO, Okuyunu K, Alagoz E, Battal B, Arslan N	An Extremely Rare Intersection: Neurolymphomatosis in a Patient with Burkitt Lymphoma Detected by 18F-Fluorodeoxyglucose Positron Emission Tomography/Computed Tomography	World Journal of Nuclear Medicine	2016	15	3	209-211	10.4103/1450-1147.172304
Öztürk E, Arpaci F, Kocaoğlu M, Arslan N, Bulakbaşi N, Özgüven M	Detection of widespread neurolymphomatosis with F-FDG PET	European Journal of Nuclear Medicine and Molecular Imaging	2006	33		975-976	10.1007/s00259-006-0146-y
Peterson J, Caliskan B, Bonyadlou S	Positron emission tomography/computerized tomography imagine of multiple focus of neurolymphomatosis	Indian Journal of Nuclear Medicine	2014	29	4	252-253	10.4103/0972-3919.142632
Ramirez-Zamora A, Morales-Vidal S, Chawla J, Biller J	Autopsy proven peripheral nervous system neurolymphomatosis despite negative bilateral sural nerve biopsy	Frontiers in Neurology	2013	4	197	1 - 3	10.3389/fneur.2013.00197
Raut T, Bhatt M, Hastak M, Shaikh I, Sanghvi D, Rabade N	Neurolymphomatosis as a presenting feature of primary testicular lymphoma	Annals of Indian Academy of Neurology	2021	24	2	269	
Strobel K, Pestalozzi B, Ciernik I, Schaefer NG, Koma AY, Hany TF	Changing PET/CT manifestation of neurolymphomatosis	European Journal of Nuclear Medicine and Molecular Imaging	2006	33	10	1244	10.1007/s00259-006-0112-8
Toledano M, Siddiqui MA, Thompson CA, Garza I, Pittock SJ	Teaching NeuroImages: Diagnostic utility of FDG-PET in neurolymphomatosis	American Academy of Neurology	2013			e3	
Trojan A, Jermann M, Taverna C, Hany TF	Fusion PET-CT imaging of neurolymphomatosis	Annals of Oncology	2002	13		802-805	10.1093/annonc/mdf057
Tsang HHC, Lee EYP, Anthony MP, Khong PL	F-FDG PET/CT Diagnosis of Vagus Nerve Neurolymphomatosis	Clinical Nuclear Medicine	2012	37	9	897-898	
Urban PP, Kaczmarek E, Wellach I, Brüning R, Brülke N, Schulte C, Knop K, Weis J			2008				10.1007/s00115-008-2456-8
Vijayan J, Chan YC, Therimadasamy A, Wilder-Smith EP	Role of combined B-mode and Doppler sonography in evaluating neurolymphomatosis	Neurology	2015	85		752-755	
Xu L, Zhou Y, Qiu D, Shams S	Fusion PET-CT detection of neurolymphomatosis originating from primary breast lymphoma: A case report and literature review	Oncology Letters	2012	4		973-975	10.3892/ol.2012.840
Yazawa S, Ohi T, Shiomi K, Takashima N, Kyoraku I, Nakazato M	Brachial Plexus Neurolymphomatosis: A Discrepancy between Electrophysiological and Radiological Findings	Internal Medicine	2007			533-534	10.2169/internalmedicine.46.6153
Bokstein F, Goor O, Shihman B, Rochkind S, Even-Sapir E, Metser U, Neufeld M	Assessment of neurolymphomatosis by brachial plexus biopsy and PET/CT. Report of a case	Journal of Neuro-Oncology	2005	72		163-167	10.1007/s11060-004-3389-0
Dong Q, Wong KK, Avram AM	Sacral Nerve Root Neurolymphomatosis Diagnosed on	Clinical Nuclear Medicine	2008	33	1	30-31	

	FDG-PET/CT and Magnetic Resonance Imaging						
Harada N, Tomita M, Kimura A, Koumura A, Hayashi Y, Hozumi I, Kanemura N, Moriwaki H, Sobue G, Inuzuka T	Case Report: Two cases of neurolymphomatosis as a manifestation of relapsed non-Hodgkin lymphoma		2012	101	1	157-160	
Kanter P, Zeidman A, Streifler J, Marmelstein V, Even-Sapir E, Mester U, Stein GY, Cohen AM	PET-CT imaging of combined brachial and lumbosacral neurolymphomatosis	European Journal of Haematology	2005	74		66-69	
Kim JH, Jang JH, Koh SB	A case of neurolymphomatosis involving cranial nerves: MRI and fusion PET-CT findings	Journal of Neuro-Oncology	2006	80		209-210	10.1007/s11060-006-9164-7
Levin N, Soffer D, Grissaru S, Aizikovich N, Gomori JM, Siegal T	Primary T-cell CNS lymphoma presenting with leptomeningeal spread and neurolymphomatosis	Journal of Neuro-Oncology	2008	90		77-83	10.1007/s11060-008-9633-2
Matsue K, Hayama BY, Iwama KI, Koyama T, Fujiwara H, Yamakura M, Takeuchi M, O'uchi T	High Frequency of Neurolymphomatosis as a Relapse Disease of Itravascular Large B-Cell Lymphoma	Cancer	2011	117		4512-4521	10.1002/cncr.26090
Murthy NK, Sharma M, Spinner RJ	Primary peripheral nerve tumors associated with nerve-territory herpes zoster	Acta Neurochirurgica	2020	162		1147-1151	10.1007/s00701-020-04292-y
Oya Y	Lymphoma in the peripheral nerves and muscles	Brain and Nerve	2014	68	8	955-967	
M Pagès, C Marty-Double, AM Pagès	Sensory Neuropathy as Revealing Symptom of Neurolymphomatosis: Report of a Case with a 15-Year Duration	European Neurology	2004	52		57-58	10.1159/000079545
Pappenheimer AM, Dunn LC, Cone V	Studies on Fowl Paralysis (Neurolymphomatosis Gallinarum)		1928			63-93	
Radnai B, Takacs-Nagy L	Orvosi hetilap		1951	92	48	1559-1562	
Shaikh F, Chan AC, Awan O, Jerath N, Reddy C, Khan SA, Graham MM	Diagnostic Yield of FDG-PET/CT, MRI, and CSF Cytology in Non-Biopsiable Neurolymphomatosis as a Heralding Sign of Recurrent Non-Hodgkin's Lymphoma	Cureus	2015	7	9	e319	10.7759/cureus.319
Shaikh F, Savells D, Awan O, Inayat F, Chaudhry A, Jerath N, Graham MM	Quantitative Imaging Analysis of FDG PET/CT Imaging for Detection of Central Neurolymphomatosis in a Case of Recurrent Diffuse B-Cell Lymphoma	Cureus	2015	7	11	e379	10.7759/cureus.379
Shima K, Ishida C, Okino S, Kotani T, Higashi K, Yamada M	A Linear Lesion along the Brachial Plexus on FDG-PET in Neurolymphomatosis	Internal Medicine	2008	47		1159-1160	10.2169/internalmedicine.47.1104
Strobel K, Fischer K, Hany TF, Poryazova R, Jung HH	Sciatic Nerve Neurolymphomatosis - Extent and Therapy Response Assessment with PET/CT	Clinical Nuclear Medicine	2007	32	8	646-648	
Swarnkar A, Fukui MB, Fink DJ, Rao GR	MR Imaging of Brachial Plexopathy in Neurolymphomatosis	AJR	1997	169		1189-1190	
Tabuchi S, Yoshioka H, Nakayasu H, Watanabe T	Primary Central Nervous System Lymphoma of the Cerebellopontine Angle That Initially	NMC Case Report Journal	2014	1		28-32	10.2176/nmccrj.2013-0353

	Occurred as Neurolymphomatosis of the Acoustic Nerve						
Talanow R, Shrikanthan S	Value of FDG PET in the Evaluation of Therapy Response in Nerve Root Neurolymphomatosis	Clinical Nuclear Medicine	2011	36	5	389-391	
Trelles JO, Trelles L	Human peripheral neurolymphomatosis	Revue neurologique	1983	139	12	703-714	
Trelles JO, Urquiaga C, Palomino L, Trelles L	Human peripheral neurolymphomatosis (reticuloendothelial polyradiculoneuritis)	Journal of the Neurological Sciences	1976	28		187-202	
van den Bent MJ, de Bruin HG, Brutel de la Rivière G, Sillevis Smitt PAE	Negative sural nerve biopsy in neurolymphomatosis	Journal of Neurology	1999	246		1159-1163	
von Falck C, Rodt T, Joerdens S, Waldeck S, Kiesel H, Knapp WH, Galanski M	F-18 2-Fluoro-2-Deoxy-Glucose Positron Emission Tomography/Computed Tomography for the Detection of Radicular and Peripheral Neurolymphomatosis	Clinical Nuclear Medicine	2009	34	8	493-495	
Islam S, Zaher A	Pathologic Quiz Case	Archives of Pathology & Laboratory Medicine	2000	124		643-644	
da Silva AN, Lopes MB, Schiff D	Rare pathological variants and presentations of primary central nervous system lymphomas	Neurosurgical Focus	2006	21	5	e7	
del Grande A, Sabatelli M, Luigetti M, Conte A, Granata G, Rufini V, del Ciello A, Gaudino S, Fernandez E, Hohaus S, Coli A, Lauriola L	Primary Multifocal Lymphoma of Peripheral Nervous System: Case Report and Review of the Literature	Muscle & Nerve	2014	50		1016-1022	10.1002/mus.24354
Draganesco S, Vuia O, Draganesco N, Seitan I, Stroia L	Landry's syndrome (neurolymphomatosis) appearing during an acute leukemia in remission	Revuew neurologique	1965	113	2	115-169	
Ghobrial IM, Buadi F, Spinner RJ, Colgan JP, Wolanskyj AP, Dyck PJ, Witzig TE, Micallef IN, O'Neill BP	High-Dose Intravenous Methotrexate Followed by Augologous Stem Cell Transplantation as a Potentially Effective Therapy for Neurolymphomatosis	Cancer	2004	100	11	2403-2407	10.1002/cncr.20263
Grisold W, Klimpfinger M, Maehr B, Pont J, Struhal W, Urbanits S, Vass A, Vesely M	Peripheral nerve involvement in lymphoma: the meninges as the crucial barrier between meningoradicular spread and neurolymphomatosis	Journal of the Peripheral Nervous System	2007	12		58-60	
He W, Wang W, Gustas C, Malysz J, Kaur D	Isolated sciatic neuropathy as an initial manifestation of a high grade B-cell lymphoma: A case report and literature review	Clinical Neurology and Neurosurgery	2016	149		147-153	10.1016/j.clineuro.2016.07.029
Kim KT, Kim SI, Do YR, Jung HR, Cho JH	Sciatic nerve neurolymphomatosis as the initial presentation of primary diffuse large B-cell lymphoma: a rare cause of leg weakness	Yeungnam University Journal of Medicine	2021	38	3	258-263	10.12701/yujm.2021.00983
Kuntzer T, Lobrinus JA, Janzer RC, Ghika J, Bogousslavsky J	Clinicopathological and Molecular Biological Studies in a	Muscle & Nerve	2000	23		1604-1609	

	Patient with Neurolymphomatosis						
Lee YC, Huang GS, Chang WC, Hsu YC	Friend eff sign: A typical ultrasonography feature of neurolymphomatosis	Journal of Clinical Ultrasound	2021			1 - 3	10.1002/jcu.23029
Mori Y, Yamamoto K. Ohno A, Fukunaga M, Nishikawa A	Primary Central Nervous System Lymphoma with Peripheral Nerve Involvement: Case Report	Cureus	2019	11	9	e5675	10.7759/cureus.5675
Mustaga R, Klein CJ, Martinez-Thompson J, Johnson AC, Engelstad JK, Spinner RJ, Crum BA	Recurrent Brachial Neuritis Attacks in Presentation of B-Cell Lymphoma	Mayo Clinic Proceedings: Innovations, Quality & Outcomes	2018	2	4	382-386	10.1016/j.mayocpiqo.2018.10.002
Nakagun S, Horiuchi N, Watanabe K, Matsumoto K, Tagawa M, Shimbo G, Kobayashi Y	CD3 and CD20 co-expression in a case of canine peripheral T-cell lymphoma with prominent cardiac and peripheral nerve involvement	Journal of Veterinary Diagnostic Investigation	2018	30	5	779-783	10.1177/1040638718794765
Padma S, Sundaram P, Praveen Kumar SLG	Primary peripheral neurolymphomatosis mimicking synovial sarcoma: FDG PETCT to the rescue	Journal of Cancer Research and Therapeutics	2014	10	3	1 - 3	
Pentsova E, Rosenblum M, Holodny A, Palomba ML, Omouro A	Chemotherapy-related magnetic resonance imaging abnormalities mimicking disease progression following intraventricular liposomal cytrabine and high dose of methoxtrexate for neurolymphomatosis	Leukemia & Lymphoma	2012	53	8	1620-1622	10.3109/10428194.2012.656632
Peruzzi P, Ray-Chaudhuri A, Slone WH, Mekhjian HS, Porcu P, Chiocca EA	Reversal of neurological deficit after chemotherapy in BCL-6-positive neurolymphomatosis	Journal of Neurosurgery	2009	111		247-251	10.3171/2008.11.JNS08291
Pietrangeli A, Milella M, de Marco S, Bartolazzi A, Motolese M, Zompetta C, Jandolo B	Brachial plexus neuropathy as unusual onset of diffuse neurolymphomatosis	Neurological Sciences	2000	21		241-245	
Rastogi P, Singh H, Gupta P, Bal A, Dutta U, Gupta N	Extra-hepatic biliary obstruction due to diffuse large B-cell lymphoma: an autopsy case report with unattended yet signification pathologies	Clinical Journal of Gastroenterology	2021	14		269-274	10.1007/s12328-020-01252-y
Shenkier TN	Unusual Variatns of Primary Central Nervous System Lymphoma	Hematology/Oncology Clinics of North America	2005			651-664	10.1016/j.hoc.2005.05.001
Suga K, Yasuhiko Kawakami, Matsunaga N, Yujiji T, Nakazora T, Ariyoshi K	F-18 FDG PET/CT Findings of a Case of Sacral Nerve Root Neurolymphomatosis that Occurred During Chemotherapy	Clinical Neuclear Medicine	2011	36	1	73-76	
Tong AKT, Neo SHS, Kok TY	Disseminated Lymphoma Evolving into Neurolymphomatosis during Mid-cylce of Chemotherapy Detected by F-FDG PET/CT	Annals Academy of Medicine	2015	44	11	545-547	
Yadav D, Angamuthu M, Subudhi TK, Das CJ, Kumar R	PET/CT in Evaluation of Low Backache Due to Scacral Nerve Root Involvement	Clinical Nuclear Medicine	2020	45	6	e299-e300	10.1097/RLU.0000000000003021
Yamada S, Tanimoto A, Nabeshima A, Tasaki T, Wang	Diffuse large B-cell lymphoma presenting with neurolyphomatosis and itravascular	Diagnostic Pathology	2012	7	1	94	10.1186/1746-1596-7-94

KY, Kitada S, Noguchi H, Sasagun Y	lymphoma: a unique autopsy case with diverse neurological symptoms						
Advani P, Paulus A, Murray P, Jiang L, Goff R, Pooley R, Jain M, Garner H, Foran J	A Rare Case of Primary High-Grade Large B-Cell Lymphoma of the Sciatic Nerve	Clinical Lymphoma, Myeloma and Leukemia	2015	15	6	e117-120	10.1016/j.clml.2014.12.001
Capek S, Hébert-Blouin MN, Puffer RC, Martinoli C, Frick MA, Amrami KK, Spinner RJ	Tumefactive appearance of peripheral nerve involvement in hematologic malignancies: a new imaging association	Skeletal Radiology	2015	44		1001-1009	10.1007/s00256-015-2151-3
Capek S, Amrami KK, Dyck PJB, Spinner RJ	Targeted fascicular biopsy of the sciatic nerve and its major branches: rationale and operative technique	Neurosurgical Focus	2015	39	3	e12	10.3171/2015.6.FOCUS15213
Cassereau J, Letournel F, François S, Dubas F, Nicolas G	Chronic inflammatory demyelinating polyneuropathy in Waldenström's macroglobulinemia	Revue Neurologique	2011	167		343-347	10.1016/j.neurol.2010.10.015
Chazot G, Berger B, Carrier H, Barbaret C, Bady B, Dumas R, Creyssel R, Schott B	Neurological manifestations in monoclonal gammopathies. Pure neurological manifestations. Immunofluorescence study	Revue Neurologique	1976	132	3	195-212	
D'Amico A, Napoli M, Cirillo M, D'Arco F, D'Anna G, Caranci F, Mariniello G, Brunetti A	Imaging of Cervical Extradural En-Plaque Meningioma	The Neuroradiology Journal	2012	25		598-603	
da Rocha AJ, Guedes BVS, da Silveira da Rocha TMB, Junior ACMM, Chiattone CS	Modern techniques of magnetic resonance in the evaluation of primary central nervous system lymphoma: contributions to the diagnosis and differential diagnosis	Revistas Brasileira de hematologia e Hemoterapia	2016	38	1	44-54	10.1016/j.bjhh.2015.12.001
Gammon B, Gammon BR, Kim YH, Kim J	Neurotropic Gamma-Delta T-Cell Lymphoma With CD30-Positive Lymphoid Infiltrates	The American Journal of Dermatopathology	2016	38	9	e133-e136	
Gemignani F, Marchesi G, Giovanni GD, Salih S, Quaini F, Nobile-Orazio E	Low-Grade Non-Hodgkin B-Cell Lymphoma Presenting as Sensory Neuropathy	European Journal of Neurology	1996	36		138-141	
Khurana A, Dalland JC, Young JR, Inwards DJ, Paludo J	Brexucabtagene autoleucel therapy induces complete remission in a primary refractory blastoid mantle cell lymphoma with neurolymphomatosis	American Journal of Hematology	2021			e298-e301	10.1002/ajh.26237
Khurana A, Novo M, Nowakowski GS, Ristow KM, Spinner RJ, Hunt CH, King RL, Lachance DH, Habermann TM, Micallef IN, Johnston PB	Clinical manifestations of, diagnostic approach to, and treatment of neurolymphomatosis in the rituximab era	Blood Advances	2021	5	5	1379-1387	10.1182/bloodadvances.2020003666
khurana	supplemental materials						
Laumonerie P, Capek S, Amrami KK, Dyck PJB, Spinner RJ	Targeted fascicular biopsy of the brachial plexus: rationale and operative technique	Neurosurgical Focus	2017	42	3	e9	10.3171/2017.1.FOCUS16404
Moshe-Lilie O, Ensrud E, Ragole T, Nizar C, Dimitrova D, Karam C	CIDP mimics: a case series	BMC Neurology	2021	21		94	10.1186/212883-021-02119-7

Noda Y, Sekiguchi K, Tokuoka H, Oda T, Hamaguchi H, Kanda F, Toda T	Ultrasonographic findings of proximal median neuropathy: A case series of suspected distal neuralgic amyotrphy	Journal of the Neurological Sciences	2017	377		1 - 5	10.1016/j.jns.2017.03.037
Scharf EL, Hanson CA, Howard MT, Keegan BM	Serial cerebrospinal fluid examinations to diagnose hematological malignancy causing neurological disease	Journal of Neuro-Oncology	2016	129		77-83	10.1007/s11060-016-2140-y
Ye BS, Sunwoo IN, Suh BC, Park JP, Shim DS, Kim SM	Diffuse Large B-Cell Lymphoma Presenting as Piriformis Syndrome	Muscle & Nerve	2010	41		419-422	10.1002/mus.21538
Yilmax S, Saier S, Yen F, Halac M	Bilateral trigeminal nerve recurrence of non-hodgkin lymphoma revealed with FDG PET/CT	Indian Journal of Nuclear Medicine	2014	29	1	1	
Albeck H, Bentzen J, Ockelmann HH, Nielsen NH, Bretlau P, Hansen HS	Familial Clusters of Nasopharyngeal Carcinoma and Salivary Gland Carcinomas in Greenland Natives	Cancer	1993	72	1	196-200	
Garcin R, Gruner J, Tinel G	Case of neurolymphomatosis in man; anatomo-clinical study	Revue neurologique	1953	88	2	81-92	
Hamaguchi M, Kokubun N, Matsuda H, Onuma H, Aoki R, Takahashi W, Mitani K, Suzuki K	A case report of secondary neurolymphomatosis showing selective nerve infiltration and massive lumbar plexus enlargement	BMC Neurology	2021	21		296	10.1186/s12883-021-02330-5
Kuhlman JJ, Moustafa MA, Gupta V, Jiang L, Tun HW	Primary Cauda Equina Lymphoma Treated with CNS-Centric Approach: A Case Report and Literature Review	Journal of Blood Medicine	2021	12		645-652	10.2147/JBM.S325264
Rahmani M, Birouk N, Amarti A, Idrissi AL, Marnissi F, Belaidi H, Faris MEA, Benchekroun S, Ouazzani R	T-cell lymphoma revealed by a mononeuritis multiplex - case report and review of literature	Revue Neurologique	2007	163	4	462-470	
Saito T, Nakahara T, Abe Y, Sugiura T, Ogata M	A 63-year-old man with progressive cauda equina/conus medullaris syndrome	Brain and Nerve	1998	50	12	1133-1141	
Umehara F, Hagiwara T, Yoshimura M, Higashi K, Arimura K	Enlarged, multifocal upper limb neuropathy with HTLV-I associated myelopathy in a patient with chronic adult T-cell leukemia	Journal of the Neurological Sciences	2008	266		167-170	10.1016/j.jns.2007.07.028
Woimant F, Schanen A, Bertail MA, Dupuy M, Lecoz P, Haguenau M, Pepin B	Myelopathy manifesting as macroglobulinemia	Annales de medecine interne	1985	136	2	121-124	
