# Supplementary figures and images for: Diagnostic Considerations for Neurolymphomatosis: A Natural History Analysis
Source: Cancers (Basel). 2026 Jun 25;18(13):2068. doi: 10.3390/cancers18132068 (PMC13359875; doi:10.3390/cancers18132068)

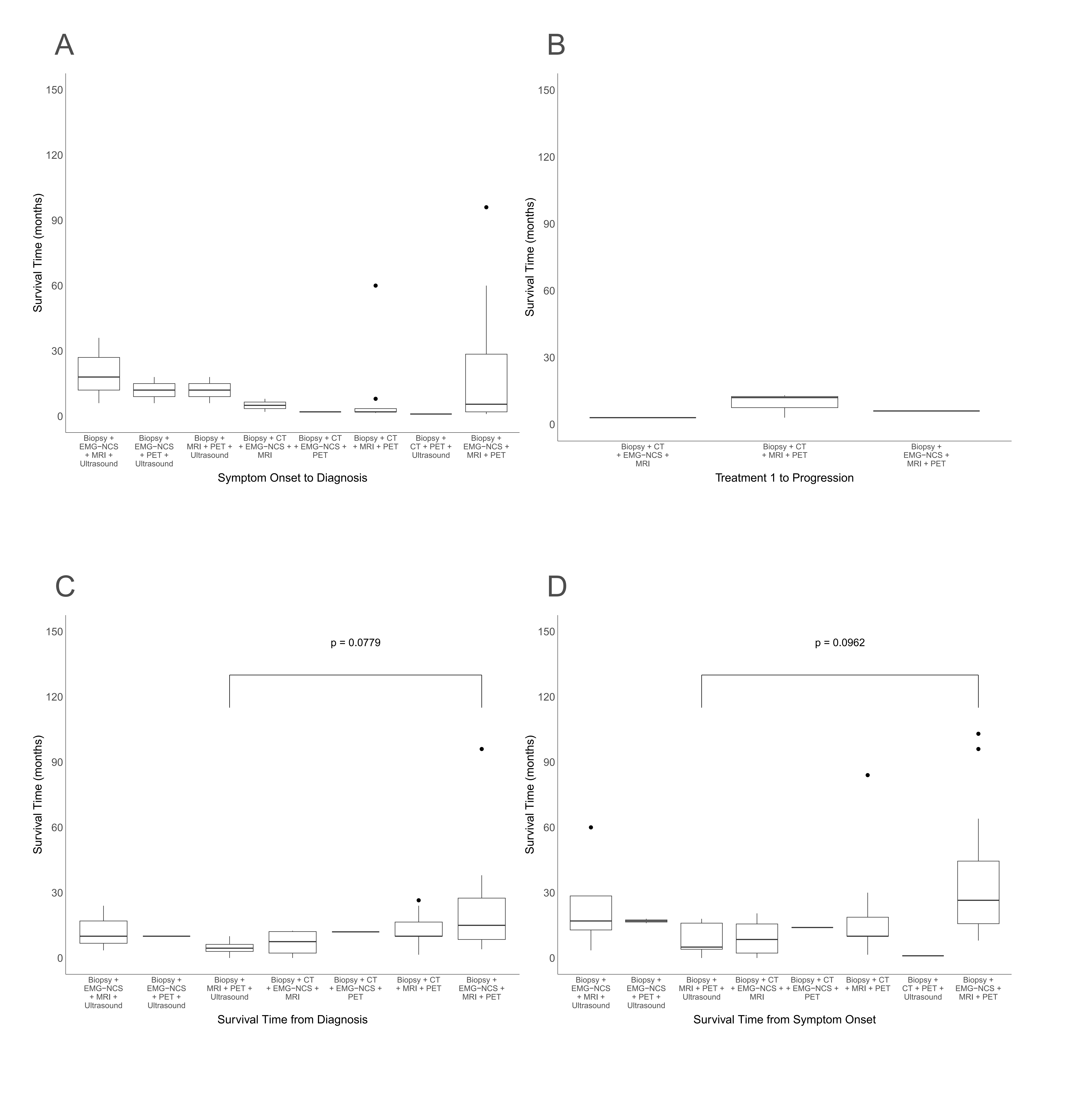

Supplement: Supplementary file 1 [file cancers-18-02068-s001.zip › Supplementary Figure S2.tif]

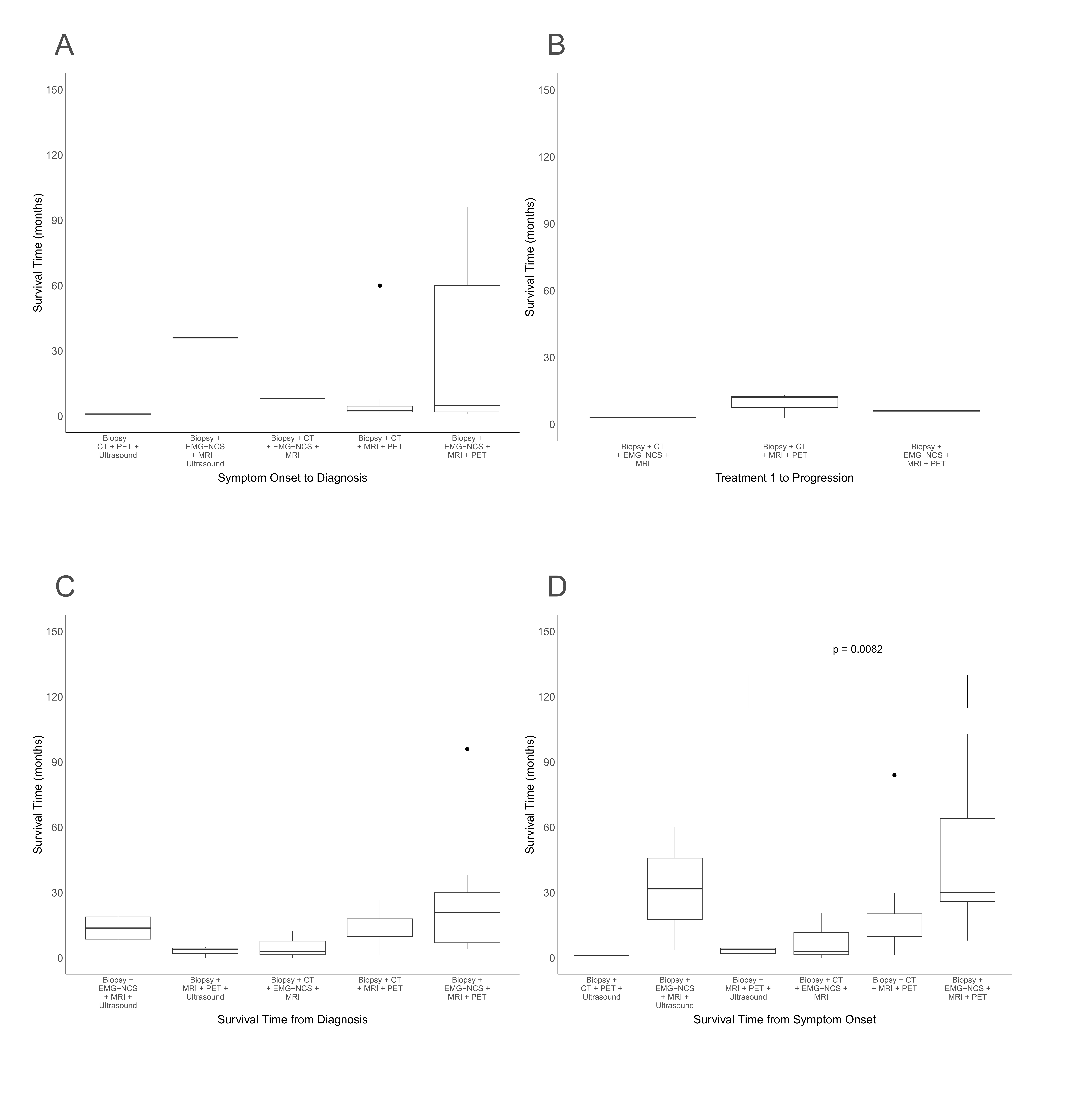

Supplement: Supplementary file 1 [file cancers-18-02068-s001.zip › Supplementary Figure S3.tif]
